# U–Pb zircon geochronology and phase equilibria modelling of HP-LT rocks in the Ossa-Morena Zone, Portugal

**DOI:** 10.1007/s00531-020-01921-w

**Published:** 2020-09-09

**Authors:** Ismay Vénice Akker, Lucie Tajčmanová, Fernando O. Marques, Jean-Pierre Burg

**Affiliations:** 1 Institute of Geological Sciences, University of Bern, Bern, Switzerland; 2 Institute of Earth Science, Heidelberg University, Heidelberg, Germany; 3 Universidade de Lisboa, 1749-016 Lisboa, Portugal; 4 Earth Sciences Department, ETH Zürich, Zürich, Switzerland

**Keywords:** Ossa-Morena zone, U–Pb geochronology, Phase equilibria modelling, High pressure-low temperature metamorphism, Cadomian basement

## Abstract

The Ossa-Morena Zone (OMZ) has a complex geological history including both Cadomian and Variscan orogenic events. Therefore, the OMZ plays an important role in understanding the geodynamic evolution of Iberia. However, the *P–T–t* evolution of the OMZ is poorly documented. Here, we combine structural and metamorphic analyses with new geochronological data and geochemical analyses of mafic bodies in Ediacaran metasediments (in Iberia known as Série Negra) to constrain the geodynamic evolution of the OMZ. In the studied mafic rocks, two metamorphic stages were obtained by phase equilibria modelling: (1) a high-pressure/low-temperature event of 1.0 ± 0.1 GPa and 470–510 °C, and (2) a medium-pressure/higher-temperature event of 0.6 ± 0.2 GPa and 550–600 °C. The increase in metamorphic temperature is attributed to the intrusion of the Beja Igneous Complex (around 350 Ma) and/or the Évora Massif (around 318 Ma). New U–Pb dating on zircons from the mafic rocks with tholeiitic affinity yields an age between 815 and 790 Ma. If the zircons crystallised from the tholeiitic magma, their age would set a minimum age for the pre-Cadomian basement. The ca. 800 Ma protolith age of HP-LT tholeiitic dykes with a different metamorphic history than the host Série Negra lead us to conclude that: (1) the HP-LT mafic rocks and HP-LT marbles with dykes were included in the Ediacaran metasediments as olistoliths; (2) the blueschist metamorphism is older than 550 Ma (between ca. 790 Ma and ca. 550 Ma, e.g., Cadomian).

**Electronic supplementary material:**

The online version of this article (10.1007/s00531-020-01921-w) contains supplementary material, which is available to authorized users.

## Introduction

Peri-Gondwanan terranes involved in the Variscan amalgamation of Europe show evidence of a late Neoproterozoic arc-related tectono-thermal history starting around 750 Ma, and can be divided into two groups: (i) Cadomian terranes, including northern Armorica, Ossa-Morena, Saxo-Thuringia, Moldanubia, and (ii) Avalonian terranes, including West Avalonia, East Avalonia, Carolina, Moravia-Silesia and NW Iberia (Murphy et al. 2006). The Cadomian terranes contain basement rocks derived from the edge of the West African Craton (Linnemann et al. 2008), and were affected by the Neoproterozoic assembly (ca. 1.0 Ga) and breakup (ca. 0.75 Ga) of the supercontinent Rodinia (Li et al. 2008; Murphy et al. 2004). From the late Neoproterozoic to the earliest Cambrian (Sánchez-Garcıa et al. 2003), the OMZ was involved in the Cadomian collision (e.g., Eguiluz et al. (2000); Linnemann et al. (2008)), which included the accretion of a continental arc (OMZ) against the passive margin of the Iberian Autochthon (Northern Gondwana). The formation of Gondwana was completed around 530 Ma (Li et al. 2008).

**Fig. 1:**
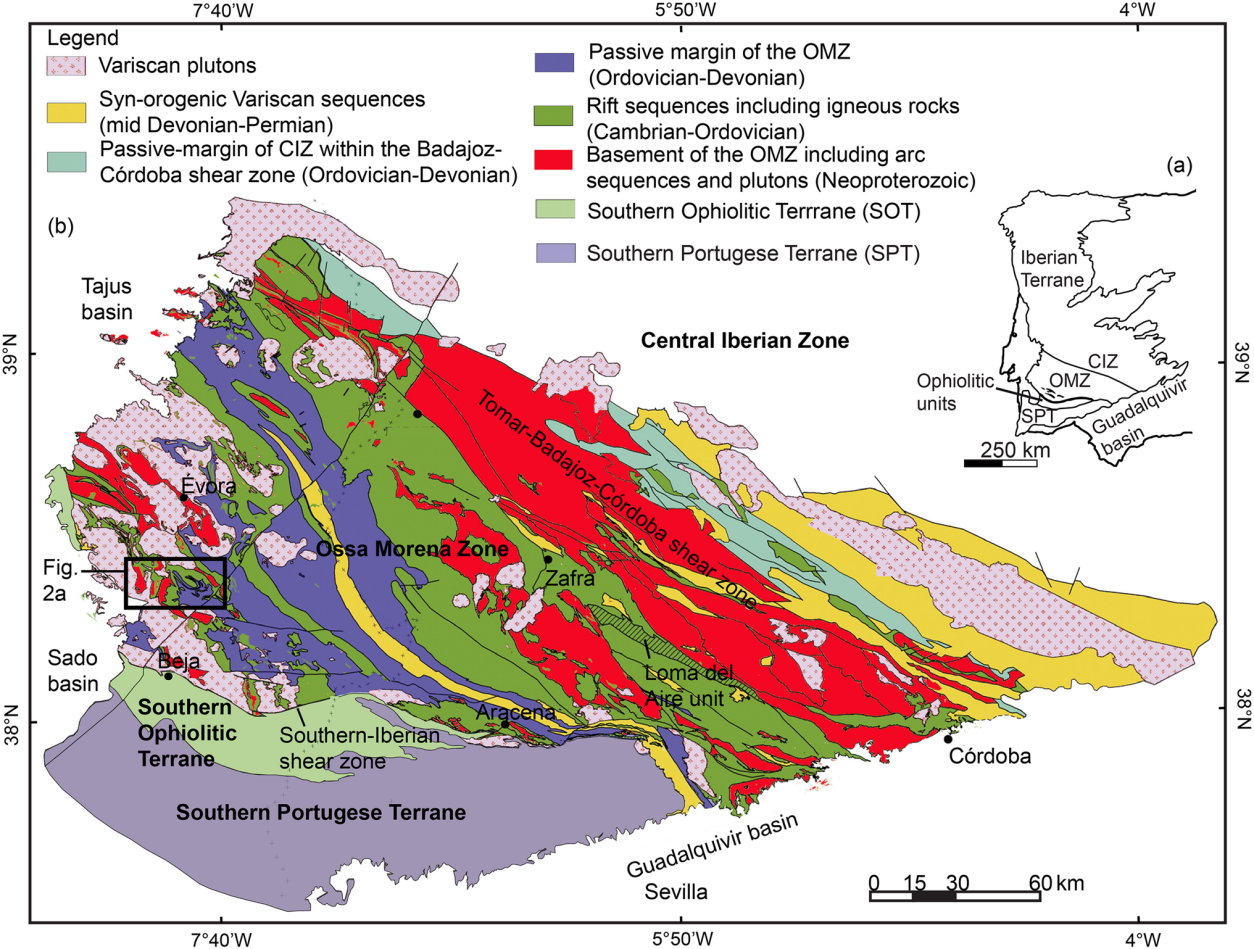
Overview of the geological terranes. **a** Main terranes are modified after Araújo et al. (2005), Ribeiro et al. (1990) and Quesada (1992). Central Iberian Zone (CIZ), Ossa-Morena Zone (OMZ), South Portuguese Terrane (SPT). **b** Detailed overview of terranes in southwestern Iberia adapted from Sánchez-García et al. (2016) and Quesada (2006). Rectangle represents the study area presented in Fig. 2a

**Fig. 2:**
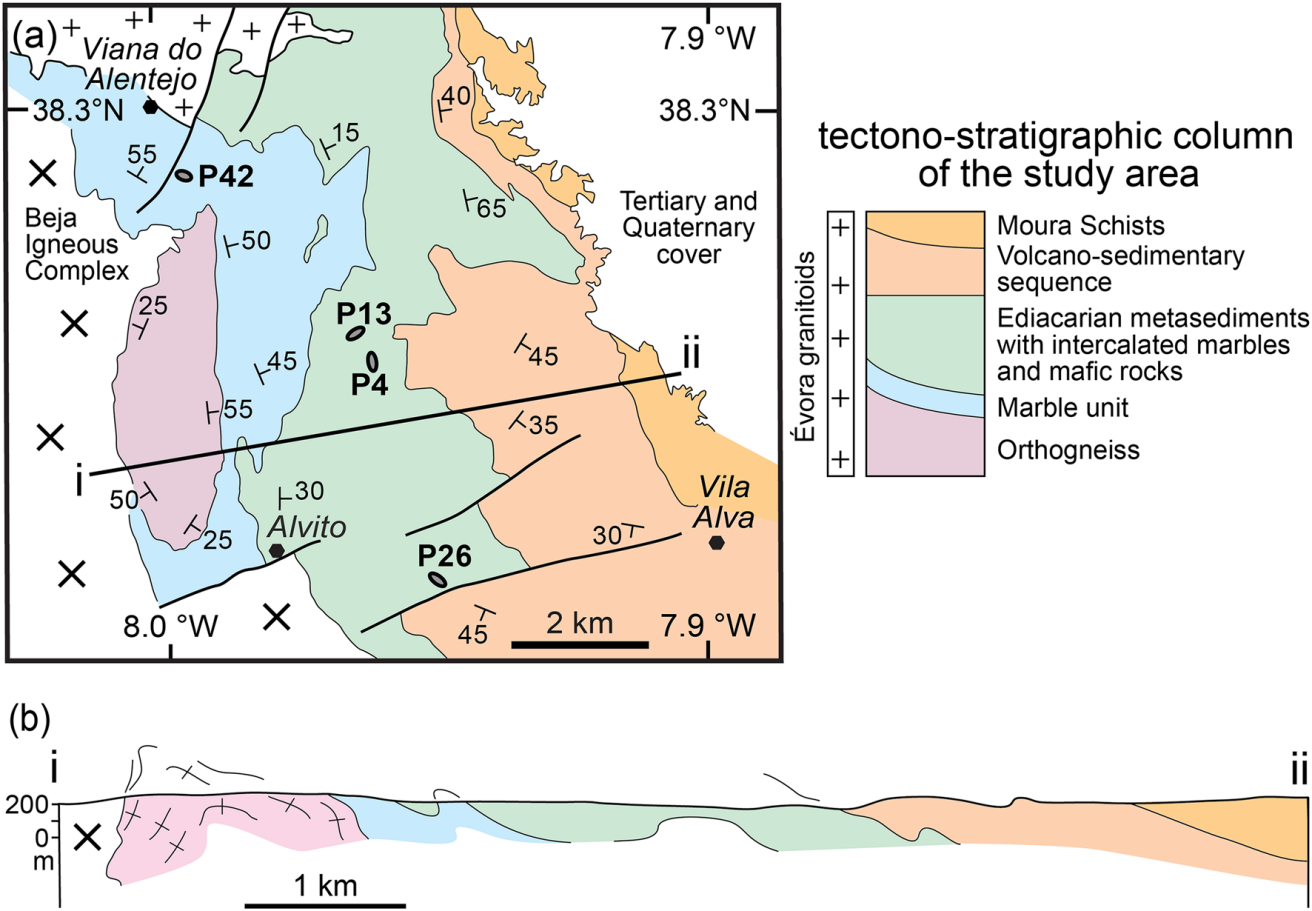
Simplified geological map and cross section of the study area compiled from Araújo et al. (2005), Oliveira et al. (1992), Rosas et al. (2008) and the Carta Geológica de Portugal na escala de 1:50.000 Folha 40-C (Viana do Alentejo). **a** Geological map with foliation symbols, cross section trace i–ii, sample locations. **b** Cross section i–ii adapted from Rosas (2003)

The protolith, geochemical signature, and *PT-t* paths of the OMZ Cadomian rocks are still poorly documented, because exposures of the basement underlying the Ediacaran metasediments (known in Iberia as Série Negra) are rare. To contribute to the understanding of the geodynamic evolution of Iberia, we focused on the OMZ area outcropping in SW Iberia (Figs. 1, 2). In this area, several questions remain controversial: (i) existence or absence of pre-Cadomian basement (> 750 Ma: Linnemann et al. (2007)); (ii) minimum age of the so-called Série Negra containing undated mafic rocks with relicts of HP-LT metamorphism (Rosas et al. 2008); (iii) the age of the HP-LT metamorphism in these mafic rocks; (iv) the meaning of the HP-LT bodies (olistoliths? tectonic slices?); and (v) correlation with, and inference of the geodynamic evolution of the OMZ on the neighboring terranes. To answer these questions, we studied two kinds of HP-LT mafic rocks: (1) mafic dykes in marbles traditionally attributed to the metasediments of the Série Negra; (2) mafic rocks in the Série Negra metasediments. The nature of the contacts between the HP-LT rocks and the Ediacaran metasediments is unspecified, because they are poorly exposed. We combined petrography, geochemistry, U–Pb zircon dating and phase equilibria modelling to characterize their protolith and metamorphic overprint. In addition, we performed U–Pb zircon dating of a granodioritic dyke intruding marbles to constrain the age relationships between the granodiorite and other magmatic bodies such as the Beja Igneous Complex (e.g., de Oliveira et al. (1991); Jesus et al. (2016); Pin et al. (2008)) and the Évora Massif (e.g., Chichorro et al. (2008); da Silva et al. (2018); Moita et al. (2009)).

## Geological setting

### Ossa-Morena Zone (OMZ)

The OMZ is bounded by two suture zones (Fig. 1), one to the north and the other to the south. The Northern Suture has been overprinted by the Tomar-Badajoz-Córdoba Shear Zone separating the OMZ from the Central Iberian Zone (e.g., Abalos et al. (1991); Pereira et al. (2010); Quesada and Dallmeyer (1994); Simancas et al. (2001)), and contains Variscan eclogites (340 ± 13 Ma) dated by U–Pb on zircon using SHRIMP (Casado 1998). Accretion of the OMZ to the Central Iberian Zone has been constrained between 540 Ma (Henriques et al. 2015) and 480 Ma (Azor et al. 2016). The Southern Suture is the Southern Iberian Shear Zone, the tectonic contact between the OMZ and the Southern Ophiolite Terrane (Crespo-Blanc and Orozco 1988; Quesada et al. 1991, 1994; Ribeiro et al. 1990).

It is currently consensual that the geodynamic evolution of the OMZ comprises three main tectonic episodes. The first episode includes an incomplete record of the Cadomian Orogeny, with the transition from a Cadomian subduction-related regime (onset of Cadomian arc magmatism dated at ca. 692 Ma by Henriques et al. (2015), and ca. 645 Ma by Sánchez-Lorda et al. (2016) to collision. The second episode comprises intracontinental rifting during the Cambrian (e.g., Abalos et al. (1991); Chichorro et al. (2008); López-Guijarro et al. (2008); Ochsner (1993); Quesada (1990); Quesada (1991); Ribeiro et al. (1990); Sánchez-Garcıa et al. (2003); Sarrionandia et al. (2012)), which culminated with the breakup of a part of Gondwana to form the Rheic Ocean (Linnemann et al. 2008; Nance et al. 2010; Quesada 1991; Sánchez-Garcıa et al. 2003). The third episode represents the Variscan cycle, in which the Late Devonian (380–360 Ma) closure of the Rheic (e.g., Nance et al. (2010)) led to the Carboniferous—Late Permian continental collision that ended with the formation of Pangaea.

**Fig. 3:**
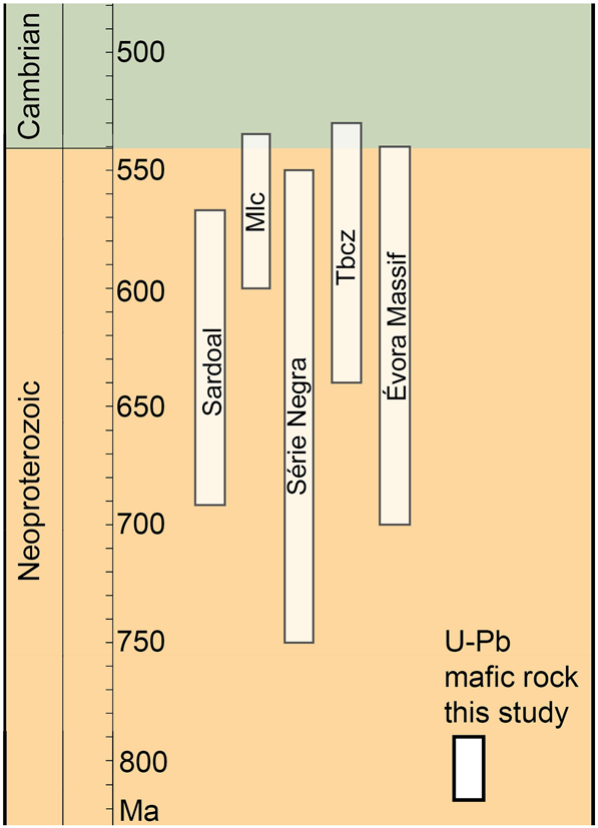
Oldest ages reported in the OMZ and the age obtained in this study. Sardoal Complex: Henriques et al. (2015); Henriques et al. (2017). Malcocinado formation (Mlc): Casado (1998); Ochsner (1993); Schäfer et al. (1993). Série Negra: Fernández-Suárez et al. (2002); López-Guijarro et al. (2008); Pereira et al. (2008); Pereira et al. (2012). Tomar-Badajoz-Córdoba Shear Zone (Tbsz): Pereira et al. (2010); Pereira et al. (2011). Évora Massif: Pereira et al. (2008)

So far, several Proterozoic to Early Paleozoic stages have been inferred (Fig. 3): (i) Formation and evolution of a Cadomian magmatic arc, the earliest of which occurred at ca. 692 Ma (Sardoal complex; Henriques et al. (2015); Henriques et al. (2017)). This was followed by the emplacement of subalkaline and peraluminous intrusions at ca. 569 Ma (Henriques et al. 2015). (ii) The formation of a metavolcano-sedimentary sequence (Malcocinado formation) dated with U–Pb on zircon between ca. 600 and 535 Ma (Casado 1998; Ochsner 1993; Schäfer et al. 1993). (iii) The deposition of Late Ediacaran rocks in the OMZ, mostly recorded in the Série Negra and overlying volcano-sedimentary sequence. (iv) The development of the Tomar-Badajoz-Córdoba Shear Zone, which includes rocks with maximum ages between ca. 640 and 530 Ma as indicated by populations of detrital zircon grains (Pereira et al. 2010, 2011). (v) The development of the Évora Massif, composed of high-grade metamorphic rocks (Moita et al. 2009; Pereira et al. 2009), with ages ranging between ca. 700 and 540 Ma inferred from populations of detrital zircons (Pereira et al. 2008) and intruded by Évora granitoids at ca. 318 Ma, dated by K–Ar (Rosas et al. 2008).

The Série Negra was originally defined in the Central Iberian Zone and subsequently extended to the OMZ (e.g., Eguiluz et al. (2000); Pereira et al. (2006)). Based on stratigraphic relationships and uncertain acritarchs (Eguiluz 1987; Quesada 1990), the Série Negra was considered a Neoproterozoic (1350–850 Ma) sedimentary sequence. Hornblende from foliated amphibolite (Obejo-Valsequillo domain in Spain) yields ^39^Ar/^40^Ar ages of ca. 550–560 Ma, which were interpreted to date cooling after Cadomian tectono-thermal activity (Dallmeyer and Quesada 1992). Schäfer et al. (1993) used zircon U–Pb provenance ages to estimate a maximum depositional age of ca. 565 Ma for the upper part of the Série Negra. A similar (560–550 Ma) upper limit of the depositional age was inferred using detrital zircon populations ca. 40 km NNW of our study area (Chichorro et al. 2008). In addition, Sm–Nd isotope data indicate that the accretion of the OMZ to the Iberian Terrane was completed by the Late Neoproterozoic–Ediacaran (ca. 540 Ma) (e.g., Fernández-Suárez et al. (2002); López-Guijarro et al. (2008); Pereira et al. (2008); Pereira et al. (2012). The Série Negra contains Paleoproterozoic (ca. 2.0 Ga) and mainly Neoproterozoic (ca. 750–550 Ma) detrital zircon populations.

### OMZ in the study area

**Fig. 4:**
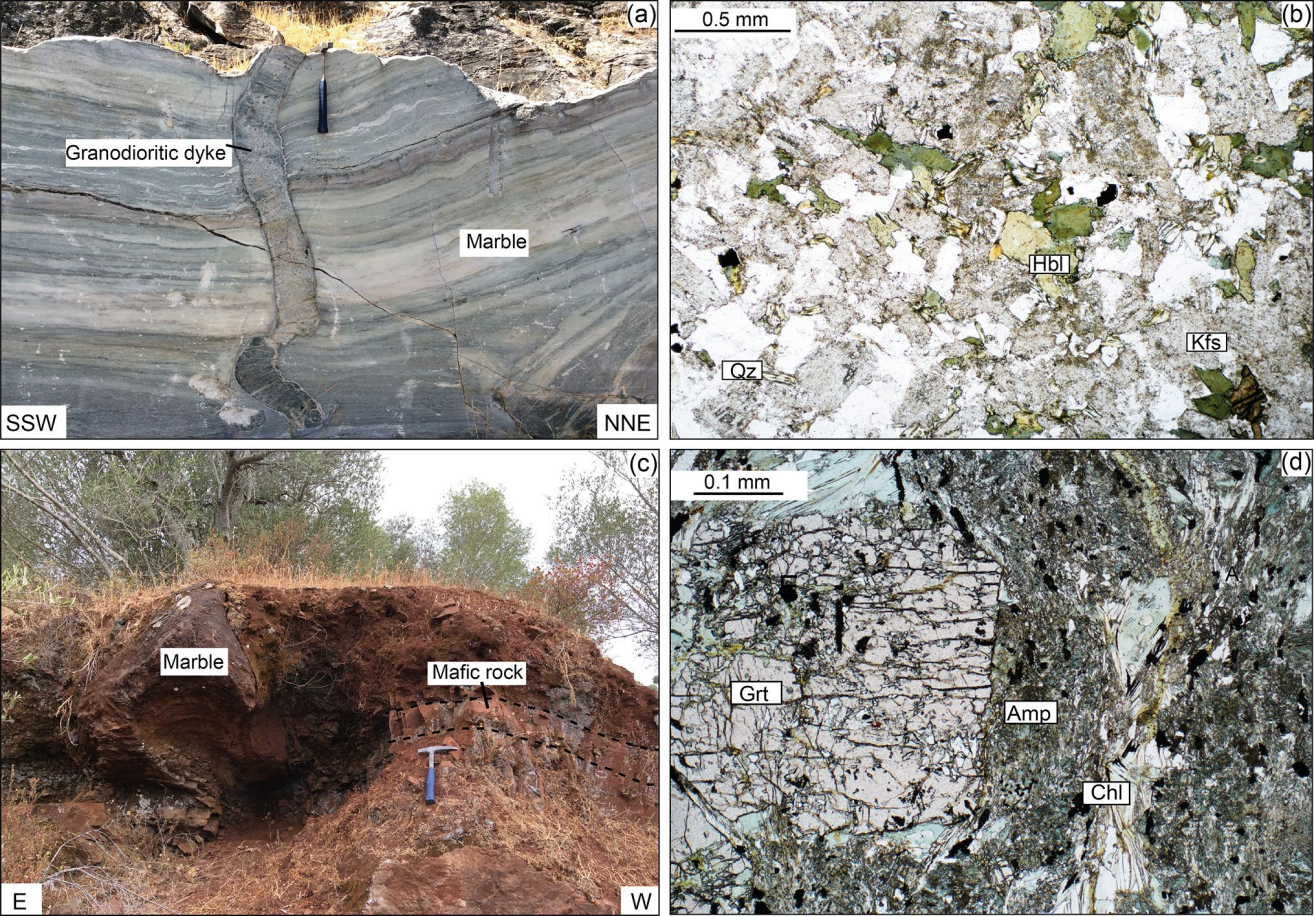
Field observations and thin section photomicrographs. **a** Granodioritic dyke in marbles (sample P42: 38° 19′30.0376″ N 8° 00′44.6220″ W). **b** Photomicrograph of granodiorite from (a). **c** Outcrop of the mafic rock in marbles (sample P26: 38° 15′05.9″ N 7° 57′05.9″ W). **d** Photomicrograph of mafic rock from **c**

According to Rosas et al. (2008) and references therein, the tectonostratigraphic sequence of SW OMZ comprises, from bottom to top (Fig. 2): (i) orthogneisses, (ii) calcite and dolomite marbles, (iii) metapelitic graphitic schists and garnet/quartz-feldspar micaschists (regionally known as Série Negra), with intercalated marbles (intruded by mafic dykes such as sample P26) and lenses of metabasic rocks (samples P4 and P13), (iv) volcano-sedimentary sequences of paragneiss and micaschist with interlayered metavolcanics and intruded metadiorites (Araújo et al. 2005), and (v) phyllites with a greenschist facies overprint, regionally known as Moura Schists (Araújo et al. 2005). In the mapped area, these formations are folded in an N-S trending antiform (Fig. 2b) and are bounded in the north by the Évora granitoids (Pereira et al. 2012; Rosas et al. 2008). Geochronology has focused on granites and gabbros close to the study area, with the Évora Massif plutonic rocks dated at ca. 340–317 Ma (Lima et al. 2012; Moita et al. 2015; Pereira et al. 2009, 2015; Rosas et al. 2008). Dallmeyer et al. (1993) reported ca. 346 and 342 Ma ^40^Ar/^39^Ar ages on hornblende from gabbros of the Beja Igneous Complex, consistent with U–Pb ages on zircon in the range 355–300 Ma (Jesus et al. 2007; Pin et al. 2008). About 10 cm to 1 m thick granodioritic dykes and sills intruded marbles close to the Viana do Alentejo village (Sample P42, Fig. 2a). These dykes are folded and boudinaged (Fig. 4a). Granodiorite dykes (Fig. 4b) cutting deformation structures in the marble suggest tectonism older than the intrusion of gabbros and diorites of the Beja Igneous Complex. Folding and boudinage of the dykes and sills, along with some structures in the marble, are possibly due to the inflation stage (ballooning) of the Beja Igneous Complex.

The tectonometamorphic evolution of the study area comprises, according to Rosas et al. (2008): (i) A high pressure-low temperature event recorded in mafic rocks contained in marbles, which suggests subduction of a continental margin. (ii) Recrystallization due to a local thermal episode, most likely induced by the Beja Igneous Complex. (iii) Top to the north shear sense in north-dipping shear zones associated with pervasive hydration and metamorphic retrogression under mostly greenschist facies, which indicates normal faulting after 358 Ma (orogenic collapse by inversion of earlier Variscan top to the S thrusts). (iv) Static recrystallization at ca. 318 Ma in hornfels around the Évora Massif granitic intrusions.

### Analytical techniques

Three representative samples of mafic rocks (samples P4, P13 and P26) and one granodioritic sample (sample P42) were chosen for detailed geochemical and petrographic analysis (sample locations in Fig. 2a; GPS coordinates in Table 1).

**Table 1:** Samples: lithology, mineral assemblage, unit and coordinates

Sample	Lithology	Mineral assemblage	Unit	Coordinates
P4	Mafic rock in micaschists and quartzites	Grt–Pl–Chl–Amp–Qz–Rt–Py	Série Negra	38° 17′ 24.0" N
				7° 58′ 12.3" W
P13	Mafic rock in micaschists and quartzites	Grt–Pl–Chl–Amp–Qz–Kfs–Ms –Rt–Py–Ap–Ilm	Série Negra	38° 17′ 35.5" N
				7° 57′ 51.1" W
P26	Mafic rock in marbles	Grt–Pl–Chl–Amp–Qz–Ep–Rt–Il m–Ttn	Série Negra	38° 15′ 05.9" N
				7° 57′ 05.9" W
P42	Granodiorite in marbles	Pl–Kfs–Qz–Hbl–Bt–Ttn	Marbles	38° 19′ 25.7" N
				8° 00′ 41.8" W

Fresh rock samples were crushed and ground in an agate mill for geochemical characterization. Bulk rock compositions were obtained using Axios XRF wavelength dispersive spectrometer from PANalytical, with an analytical error of around 1–2% (Online Resource Table S.1). Trace elements, REEs, LILEs and HFSEs were analysed by an Elan ICP-MS 6100 DRC of Perkin Elmer and a Geolas laser system at the Institute of Geochemistry and Petrology of the ETH-Zürich. Detection limits and uncertainties for these elements are given in the Online Resource (Table S.2) and show a 1*σ* of about 1–2 ppm for the trace elements with low limits of detection (LOD).

Mineral compositions were obtained with a Jeol JXA-8200 electron microprobe at the Institute of Geochemistry and Petrology of the ETH-Zürich. An acceleration voltage of 15 kV and a beam current of 22.7 nA with a defocused beam of 2 µm in diameter was used for the analyses, except for feldspar which was measured using a defocused beam with a diameter of 10 µm. Peaks and background noise were measured 20 s for each element except for Na and K, for which both peak and background measuring time were 10 s. For measurements, wollastonite (Si and Ca), albite (Na), corundum (Al), forsterite (Mg), fayalite (Fe), rutile (Ti), pyrolusite (Mn), chromite (Cr) and phlogopite (K), albite (Si and Na) and anorthite (Ca and Al) were used as natural and synthetic standards.

For U–Pb dating, additional sample material (sample P26, P42) was crushed using SelFrag apparatus and sieved in the first stage of mineral separation using a 250 μm mesh. At a second stage, magnetic separation techniques and later heavy liquids were used before handpicking of zircons using a binocular microscope, and mounting in epoxy resin and polishing. A scanning electron microscope (SEM) FEI Quanta 200f with Energy-dispersive X-ray spectroscopy (EDX) with a backscatter electron detector (BSED) and cathodoluminescence (CL) detector was used to visualize the internal structure of the zircons. All these analyses were carried out at the Scientific Center for Optical and Electron Microscopy (ScopeM) at ETH-Zürich. An ASI (Resonetics) Resolution S155 laser ablation system was used in combination with a Thermo Element XR, Sector-field single collector ICP-MS instrument. Both systems were provided by the Department of Earth Sciences, ETH-Zürich. All measurements were carried out with a spot size of 30 µm. GJ-1 (Jackson et al. 2004) was used as primary reference material, Temora (Black et al. 2003) and 91,500 (Wiedenbeck et al. 1995) as secondary reference material. For the data analysis, Iolite 2.5 processing package was used and VizualAge software package and Isoplot from the Berkeley Geochronology Center for uncertainty propagation and age calculation, assuming that the reference material and the samples behave identically (Ludwig 2003; Paton et al. 2011; Petrus and Kamber 2012).

### Petrography, mineral chemistry and whole-rock geochemistry

**Fig. 5:**
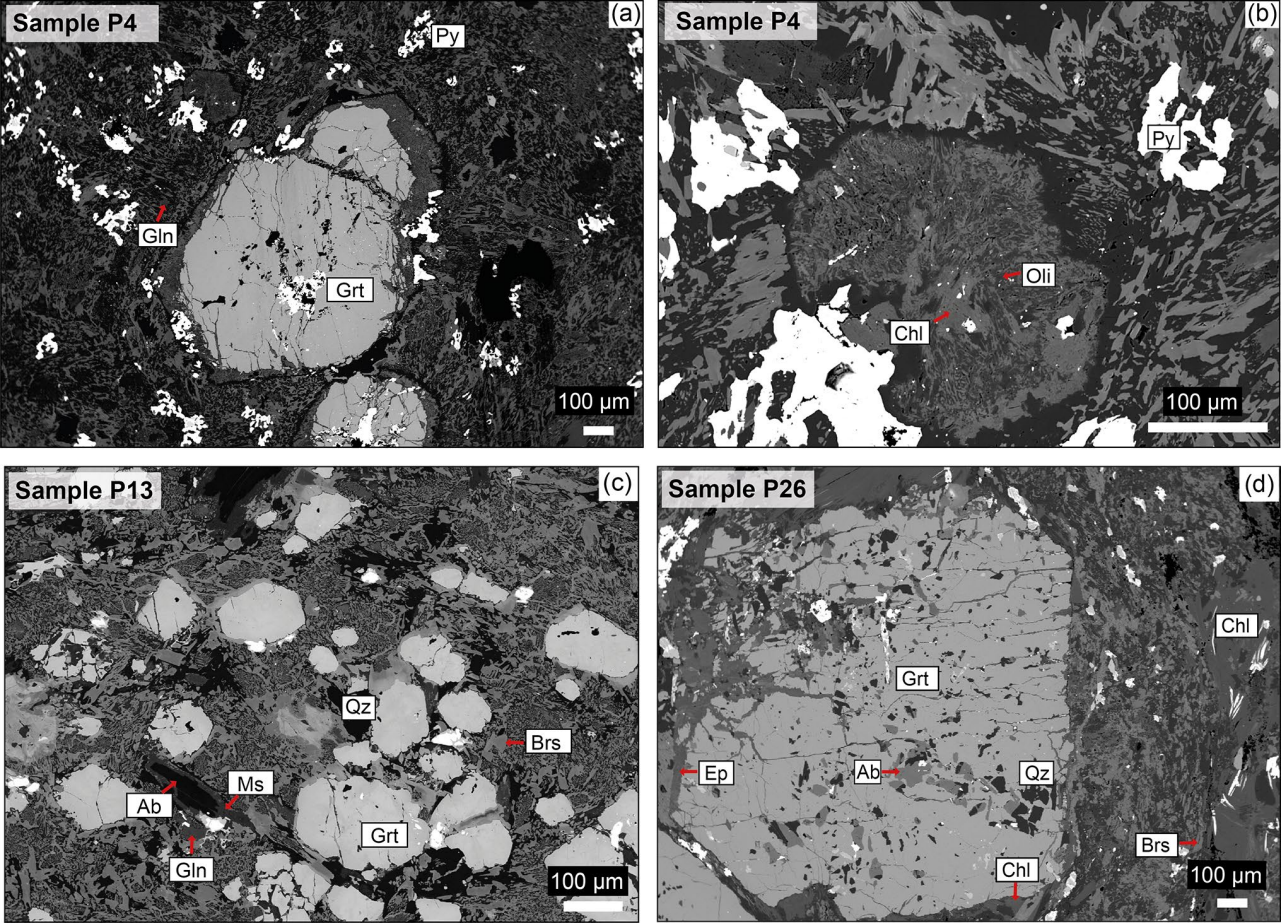
Backscatter electron (BSE) images showing mineralogy, rock microstructure and mineral replacement. **a** Garnet porphyroblasts in fine-grained matrix (sample P4). **b** Garnet replaced by chlorite and plagioclase (sample P4). **c** Garnet, with small number of inclusions, in fine-grained matrix (sample P13). **d** Garnet with many inclusions in fine-grained matrix (sample P26)

Two samples (P4 and P13) were collected from mafic lenses in the Série Negra micaschists and quartzites. The third sample (P26) is a mafic rock in marbles (Fig. 4c, d). The texture and main mineralogy of the three mafic samples are characterized by large garnet porphyroblasts in a fine-grained matrix of feldspar, chlorite, amphibole, quartz and minor pyrite, apatite and titanite (Fig. 5). Additionally, sample P13 contains muscovite and sample P26 contains epidote. We also sampled a granodioritic dyke in marble (P42; Fig. 4a, b) to obtain geochronological data.

Mineral assemblages of the mafic and the granodiorite samples are given in Table 1. The mineral compositions of garnet, feldspar and amphibole are given in Table 2. Mineral abbreviations are after Whitney and Evans (2010). The detailed textures, chemistry and *P–T* estimates of the samples are described below.

**Table 2:** Mineral compositions of garnet, feldspar and amphibole from mafic rocks

Mineral	Grt	Grt	Grt	Grt	Grt	Grt	Pl	Pl	Pl	Pl	Kfs	Pl	Amp	Amp	Amp	Amp	Amp
Sample	P4	P4	P13	P13	P26	P26	P4	P4	P13	P13	P13	P26	P4	P13	P13	P13	P26
Comment	rim	core	rim	core	rim	core	Oli	Ab	Oli	Ab	Or	Ab	Gln	Gln	Mhb	Brs	Brs
SiO_2_	37.71	37.47	36.44	37.26	37.70	37.17	60.40	65.71	61.62	65.46	61.12	66.89	52.75	54.85	44.86	46.72	48.49
Al_2_O_3_	21.56	21.32	21.67	21.35	21.59	21.07	23.57	20.87	24.75	20.25	19.47	20.09	11.97	11.62	10.44	9.71	8.77
TiO_2_	0.15	0.21	0.12	0.19	0.07	0.21	0.06	0.03	0.03	0.04	0.05	0.07	0.07	0.22	0.11	0.17	0.17
Cr_2_O_3_	0.00	0.00	0.00	0.00	0.00	0.01	0.00	0.00	0.00	0.00	0.00	0.00	0.00	0.00	0.00	0.00	0.00
MgO	2.61	1.10	2.90	1.06	2.51	1.50	0.50	0.03	0.00	0.22	0.00	0.01	11.47	11.36	12.67	12.98	11.67
FeO	28.28	24.45	27.41	25.27	30.81	30.61	0.64	0.23	0.38	0.37	0.22	0.38	8.45	8.47	13.52	11.87	15.83
MnO	0.13	6.17	0.22	1.91	0.60	2.90	0.00	0.00	0.00	0.00	0.03	0.00	0.01	0.05	0.11	0.12	0.13
CaO	10.07	9.99	10.32	12.72	8.10	8.13	3.61	0.49	5.44	0.82	0.00	0.78	1.85	0.78	9.69	9.15	8.72
Na_2_O	0.03	0.00	0.02	0.02	0.03	0.02	8.73	10.87	8.17	10.70	0.07	10.77	6.15	5.80	1.59	1.81	2.43
K_2_O	0.00	0.01	0.00	0.00	0.02	0.00	0.04	0.02	0.06	0.05	16.23	0.04	0.03	0.02	0.29	0.22	0.12
Si	2.97	2.98	2.92	2.97	2.97	2.96	2.74	2.93	2.72	2.93	2.92	2.96	7.64	7.83	6.85	7.07	7.18
Al	2.00	2.00	2.04	2.01	2.00	1.97	1.26	1.10	1.29	1.07	1.10	1.05	2.04	1.96	1.88	1.73	1.53
Ti	0.01	0.01	0.01	0.01	0.00	0.01	0.00	0.00	0.00	0.00	0.00	0.00	0.01	0.02	0.01	0.02	0.02
Cr	0.00	0.00	0.00	0.00	0.00	0.00	0.00	0.00	0.00	0.00	0.00	0.00	0.00	0.00	0.00	0.00	0.00
Mg	0.31	0.13	0.35	0.13	0.29	0.18	0.03	0.00	0.00	0.01	0.00	0.00	2.48	2.42	2.88	2.93	2.58
Fe^tot^	1.86	1.63	1.84	1.68	2.03	2.04	0.02	0.009	0.014	0.01	0.01	0.01	1.02	1.01	1.73	1.50	1.96
Mn	0.01	0.42	0.02	0.13	0.04	0.20	0.00	0.00	0.00	0.00	0.00	0.00	0.00	0.01	0.01	0.02	0.02
Ca	0.85	0.85	0.89	1.09	0.68	0.69	0.18	0.02	0.26	0.04	0.00	0.04	0.29	0.12	1.58	1.48	1.38
Na	0.00	0.00	0.00	0.00	0.01	0.00	0.77	0.94	0.70	0.93	0.01	0.92	1.73	1.61	0.47	0.53	0.70
K	0.00	0.00	0.00	0.00	0.00	0.00	0.00	0.00	0.00	0.00	1.00	0.00	0.01	0.00	0.06	0.04	0.02
total	8.02	8.01	8.05	8.02	8.03	8.05	5.01	5.00	4.98	5.00	5.03	4.98	15.20	14.97	15.47	15.33	15.39
Fe^3+^	0.06	0.03	0.16	0.05	0.09	0.14											
Fe^2+^	1.80	1.59	1.66	1.63	1.93	1.89											
*X*_Mg_	0.14	0.07	0.16	0.07	0.13	0.08											
*X*_An_							0.19	0.02	0.27	0.04	0.00	0.04					
*X*A_lm_	0.62	0.54	0.60	0.56	0.67	0.66											
*X*_Prp_	0.10	0.04	0.11	0.04	0.10	0.06											
*X*_Sps_	0.00	0.14	0.00	0.04	0.01	0.06											
*X*_Gros_	0.28	0.28	0.29	0.36	0.22	0.22											

Number of ions for garnet, feldspar and amphibole on basis of 12, 8 and 23 oxygen atoms, respectively. Mineral abbreviations after Whitney and Evans (2010); Oli is oligoclase. *X*_Alm_ = Fe/(Ca + Fe + Mg + Mn), *X*_Prp_ = Mg/(Ca + Fe + Mg + Mn), *X*_Sps_ = Mn/(Ca + Fe + Mg + Mn), *X*_Gros_ = Ca/(Ca + Fe + Mg + Mn), *X*_Mg_ = Mg/(Fe + Mg), *X*_An_ (Ca/(Ca + Na)

## Detailed petrographic description

### Mafic sample P4

**Fig. 6:**
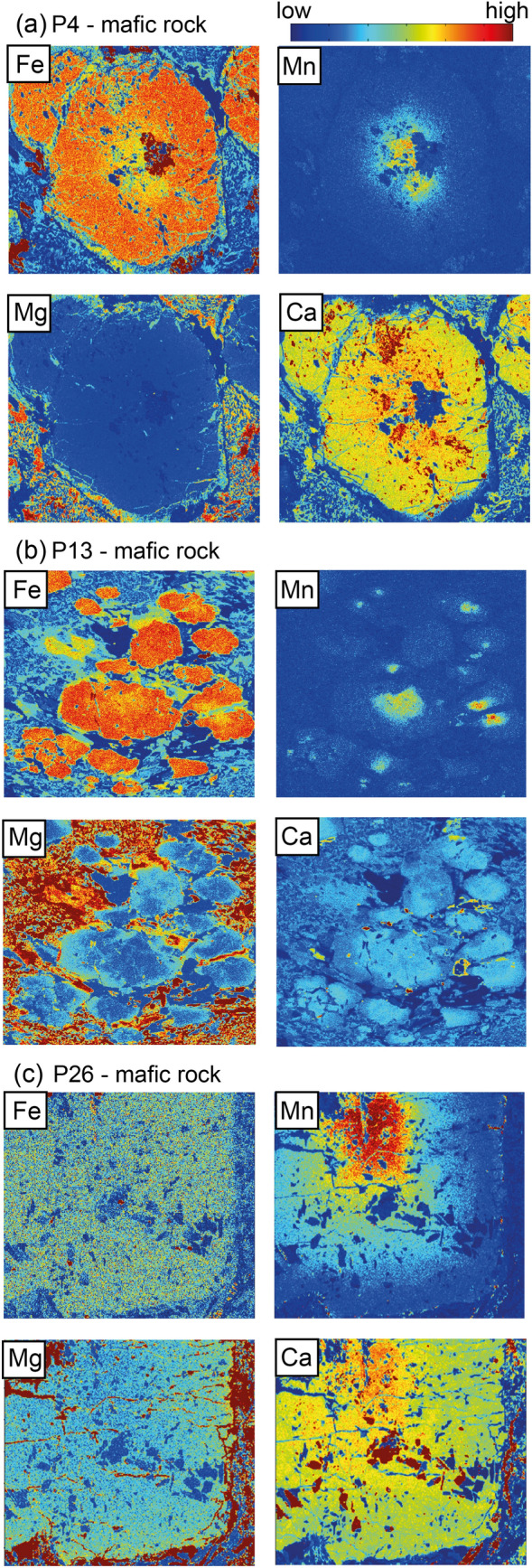
Element maps (Fe, Mn, Mg and Ca) in garnet from **a** sample P4; **b** sample P13 and **c** sample P26. Note the patchy asymmetric distribution in the garnet rims in sample P13 (**b)**, best visible in the Mg map. The Mg-rich areas occur in the elongated parts of the garnet grains

**Fig. 7:**
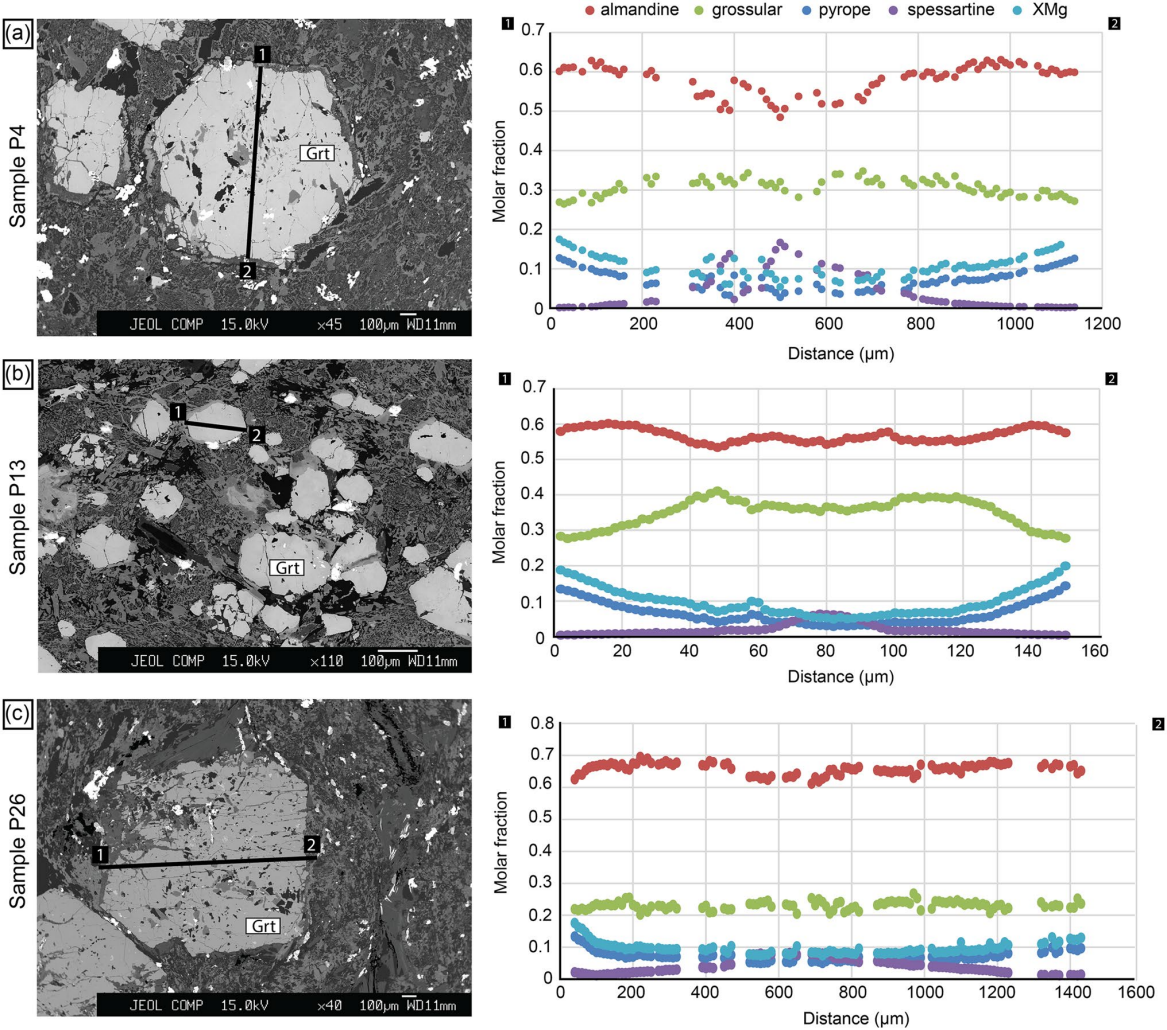
Compositional profiles showing chemical zonation in garnet from **a** sample P4; **b** sample P13 (the chemical profile goes across the elongated direction of garnet with patchy areas visible in Fig. 6b) and **c** sample P26

Garnet porphyroblasts are around 1 mm in size and contain inclusions of quartz, albite, amphibole, ilmenite, rutile and iron oxide. In some cases, the garnet porphyroblasts are replaced by chlorite and plagioclase, resulting in a symplectitic texture (Fig. 5a, b). Garnets are almandine-rich. The element mapping and compositional profiles show chemically zoning. The garnet cores are characterized by Alm_54_Gros_28_Prp_4_Sps_14_ and *X*_Mg_ = 0.05–0.08. The rims correspond to Alm_61_Gros_28_Prp_10_Sps_<1_ and *X*_Mg_ = 0.12–0.14 (Table 2; Figs. 6a, 7a). Feldspar grains are chemically homogeneous. Two plagioclase compositions were measured: albite and oligoclase (Table 2). The albitic plagioclase occurs as euhedral grains in the matrix. The oligoclase grains with *X*_An_ = 0.19 are intergrowths with chlorite in the matrix and occasionally replacing garnet (Fig. 5b). Amphibole grains are 10–20 µm in size. The most abundant amphibole is glaucophane relics in the matrix overgrown by the calcic and sodic-calcic amphiboles (Tables 2, 3). Mg-hornblende, taramite, actinolite and Fe-pargasite are minor components. The calcic and sodic-calcic amphiboles are the main phases in the fine-grained matrix.

**Table 3:** The most abundant amphibole composition in the mafic rock samples

	Calcic	Sodic-calcic	Sodic
Sample	Mhb	Brs	Gln
P4			v
P13	v	v	v
P26		v	

Classification from Leake et al. (1997): calcic, sodic-calcic and sodic amphiboles

### Mafic sample P13

Garnet porphyroblasts are dominantly elliptical with width of 50 µm and length of 200 µm. They contain a few pyrite inclusions. Garnets are almandine rich and chemically zoned with Alm_56_Gros_28_Prp_4_Sps_4_ and *X*_Mg_ = 0.05–0.07 in the core and Alm_60_Gros_29_Prp_11_Sps_<1_ and *X*_Mg_ = 0.12–0.16 in the rim (Table 2; Figs. 6b, 7b). The rims have locally asymmetric chemical distributions (Fig. 6b). Two types of plagioclase grains (albitic and oligoclase) were found (Table 2). The oligoclase has *X*_An_ = 0.27–38. In some cases, albite is surrounded by a muscovite rim with a negligible celadonite component (Fig. 5c). Additionally, sample P13 contains minor K-feldspar. Amphibole grain size is 10–20 µm. The most abundant amphiboles are glaucophane, Mg-hornblende and barroisite (Tables 2, 3). Edenite and Fe-pargasite are minor components. Glaucophane relics are overgrown by the calcic and sodic-calcic amphiboles (Fig. 5c), which are the main phases in the fine-grained matrix.

### Mafic sample P26

Garnet porphyroblasts are 1–1.2 mm in diameter. Garnet contains many inclusions of quartz, albite, epidote, ilmenite, rutile and magnetite. The pressure fringes around garnet porphyroblasts are composed of chlorite, epidote and feldspar. Garnet is chemically zoned with garnet cores of Alm_66_Gros_22_Prp_6_Sps_6_ and *X*_Mg_ = 0.07–0.08 and garnet rims of Alm_67_Gros_22_Prp_10_Sps_1_ *X*_Mg_ = 0.10–0.13 (Table 2; Figs. 6c, 7c). In this sample, only the albitic plagioclase was observed. Amphibole grains are 10–20 µm in size and their composition corresponds mostly to barroisite and minor Mg-hornblende (Tables 2, 3).

### Whole-rock geochemistry

The whole-rock geochemistry of the studied mafic rocks is required for the phase equilibria modelling. The results are given in the Online Resource (Tables S.1, S.2). Major element compositions yield SiO_2_ values of 49–50 wt%, FeO from 12–14 wt%, Al_2_O_3_ from 13 to 15 wt%, MgO around 7 wt%, CaO from 8 to 9 wt%, Na_2_O from 3 to 4 wt%, MnO from 0.1 to 0.2 wt% and TiO_2_ from 2 to 3 wt%. The Mg number (*X*_Mg_ = molar MgO/(MgO + FeO)) varies from 0.51 in sample P4 to 0.53 in sample P13 and 0.48 in sample P26.

**Fig. 8:**
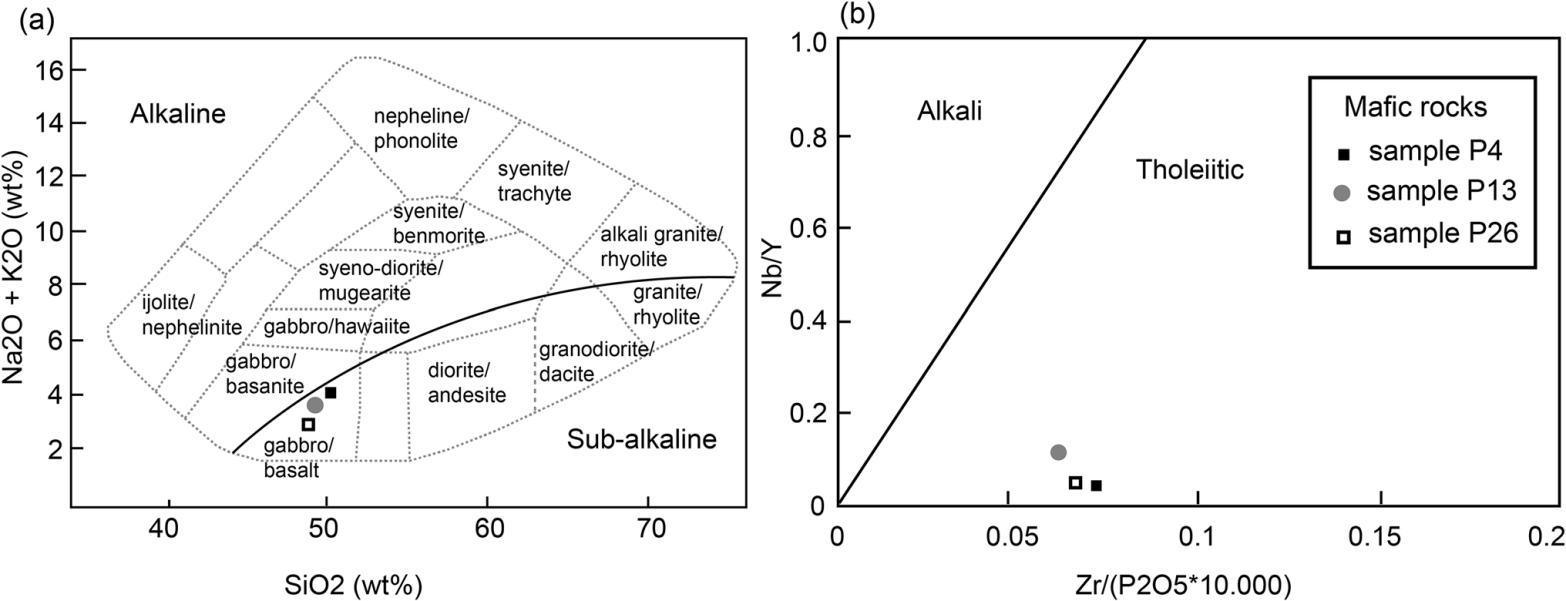
Geochemical discrimination diagrams of mafic rocks. **a** Compositions plot in the total alkalis vs. silica diagram in the gabbro/basalt field. Diagram from Wilson (1989). Black line divides alkaline (above) from sub-alkaline (below) compositions (Miyashiro 1978). **b** Immobile element diagram Nb/Y vs. Zr/P_2_O_5_ shows a tholeiitic affinity for all three samples. Diagram from Floyd and Winchester (1975)

Plotting the SiO_2_ vs. the total alkali content (Fig. 8a) defines a basaltic-gabbroic protolith with a sub-alkaline to moderately alkaline affinity. Immobility of major elements is expected (e.g., Gómez-Pugnaire et al. (2003)), and therefore, the geochemical compositions are also presented in a Nb/Y vs. Zr/P_2_O_5_ diagram (Fig. 8b). This diagram shows that the three mafic rocks have Nb/Y ratios around 0.07 and Zr/P_2_O_5_ ratios sbetween 0.06 and 0.07, indicating a tholeiitic affinity (Floyd and Winchester 1975).

**Fig. 9:**
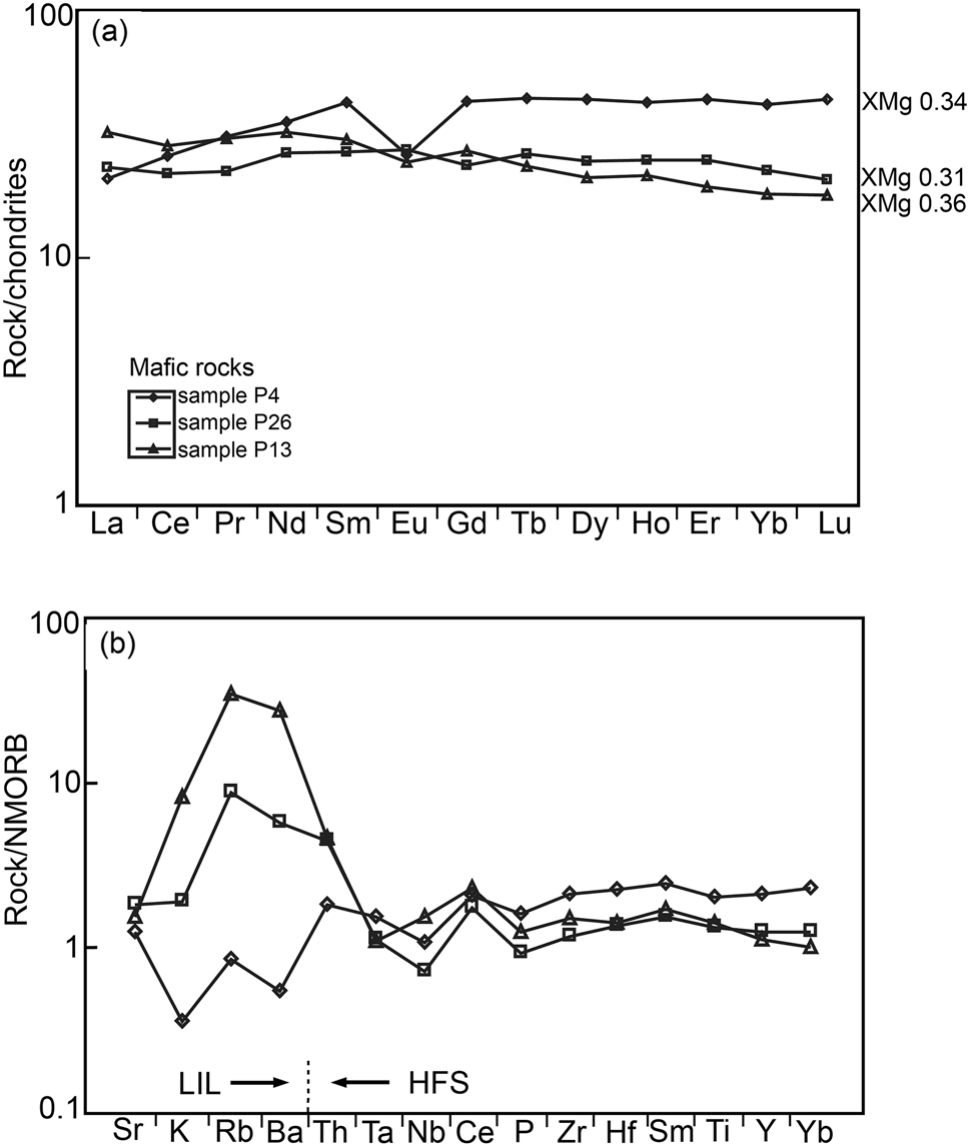
Trace element compositions and REE of the mafic rocks normalized after Sun and McDonough (1989). **a** Compositions of the REE. **b** The compositions of the LIL and HFS elements with arrows indicating increasing compatibility

The rare earth elements (REE) in samples P26 and P13 follow almost parallel patterns with a slight enrichment in the LREE (Fig. 9). Both trace element and REE diagrams of sample P4 differ from the other two samples. Mostly, the LIL elements show a deviating trend, and the REE show an increase towards the less incompatible elements and a strong Eu depletion. In both samples, P26 and P13, La and Ce are depleted, probably because of the extraction of apatite (Gómez-Pugnaire et al. 2003). Sample P26 shows a small positive Eu anomaly, whereas sample P13 shows a weak Eu depletion.

### Phase equilibria modelling

Phase equilibria modelling was done using the Perple_X software package, based on Gibbs energy minimization for a system with a specified bulk rock composition (Connolly 2009). The representative chemical system for samples P4 and P13 was MnO–Na_2_O–CaO–FeO–MgO–Al_2_O_3_–SiO_2_–H_2_O (MnNCFMASH), assuming water in excess for the considered sub-solidus conditions. Given that sample P26 contains epidote, ferric iron was considered in the modelled chemical system MnO–Na_2_O–CaO–FeO–MgO–Al_2_O_3_–SiO_2_–H_2_O–O_2_ (MnNCFMASHO) with water in excess. Involving TiO_2_ as a minor component in the calculation did not provide realistic results for the observations. Therefore, TiO_2_ was not considered in the final diagrams. Calculations were done using the thermodynamic database of Holland and Powell (1998) as revised in 2002. The solution model for garnet was from Holland and Powell (1998), plagioclase from Newton et al. (1980), amphibole from Dale et al. (2005) (for samples P4 and P13), amphibole and actinolite from Massonne and Willner (2008) (for ferric iron-bearing system in sample P26), ilmenite and magnetite from Andersen and Lindsley (1988) and omphacite from Holland and Powell (1996). Quartz was considered as a pure phase. The change in solution models in P26 results in a different topology of the *P–T* section and the stability fields of the phases. *P–T* conditions were inferred using garnet and plagioclase composition by plotting *X*_Mg_ of garnet and *X*_An_ of plagioclase. Additionally, *X*_Gros_ compositional isopleths were used for pressure estimates in sample P26 due to the missing plagioclase with oligoclase composition.

**Fig. 10:**
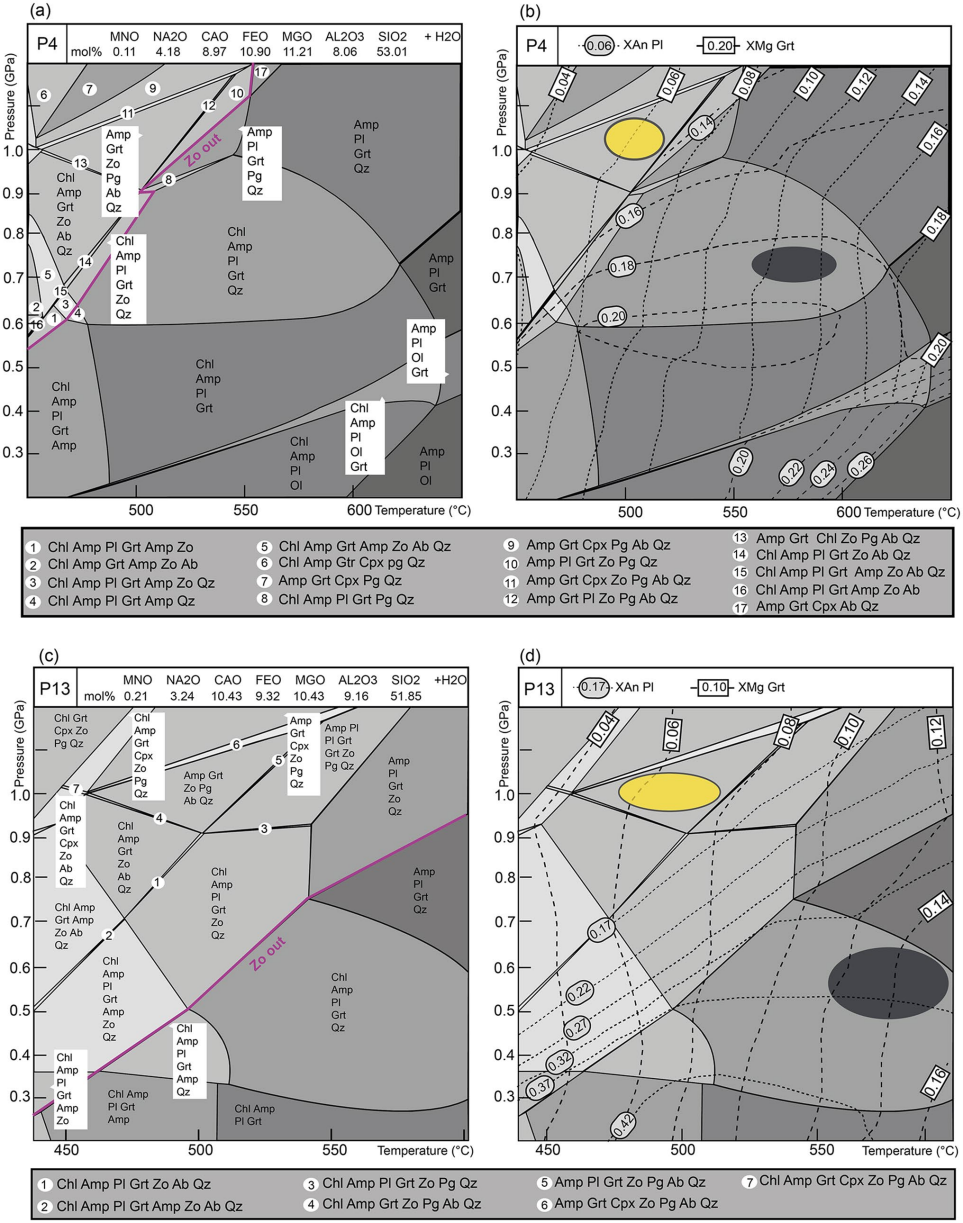
Phase diagram sections and compositional isopleths for sample P4 and P13. **a** Phase diagram section of sample P4 calculated in the MnNCFMASH system with H_2_O in excess. **b** Isopleths of X_Mg_ in garnet and X_An_ of plagioclase for sample P4. **c** Phase diagram section of sample P13 calculated in the MnNCFMASH system with H_2_O in excess. **d** Isopleths of X_Mg_ in garnet and X_An_ of plagioclase for sample P13. The purple line indicates the appearance of zoisite, which coincides with a drop in the X_An_ of plagioclase. Yellow ellipse represents *P–T* condition of high-pressure event inferred from the garnet core composition and albitic plagioclase. Dark ellipse represents *P–T* condition of lower pressure re-equilibration using garnet rim composition and oligoclase from the matrix

**Fig. 11:**
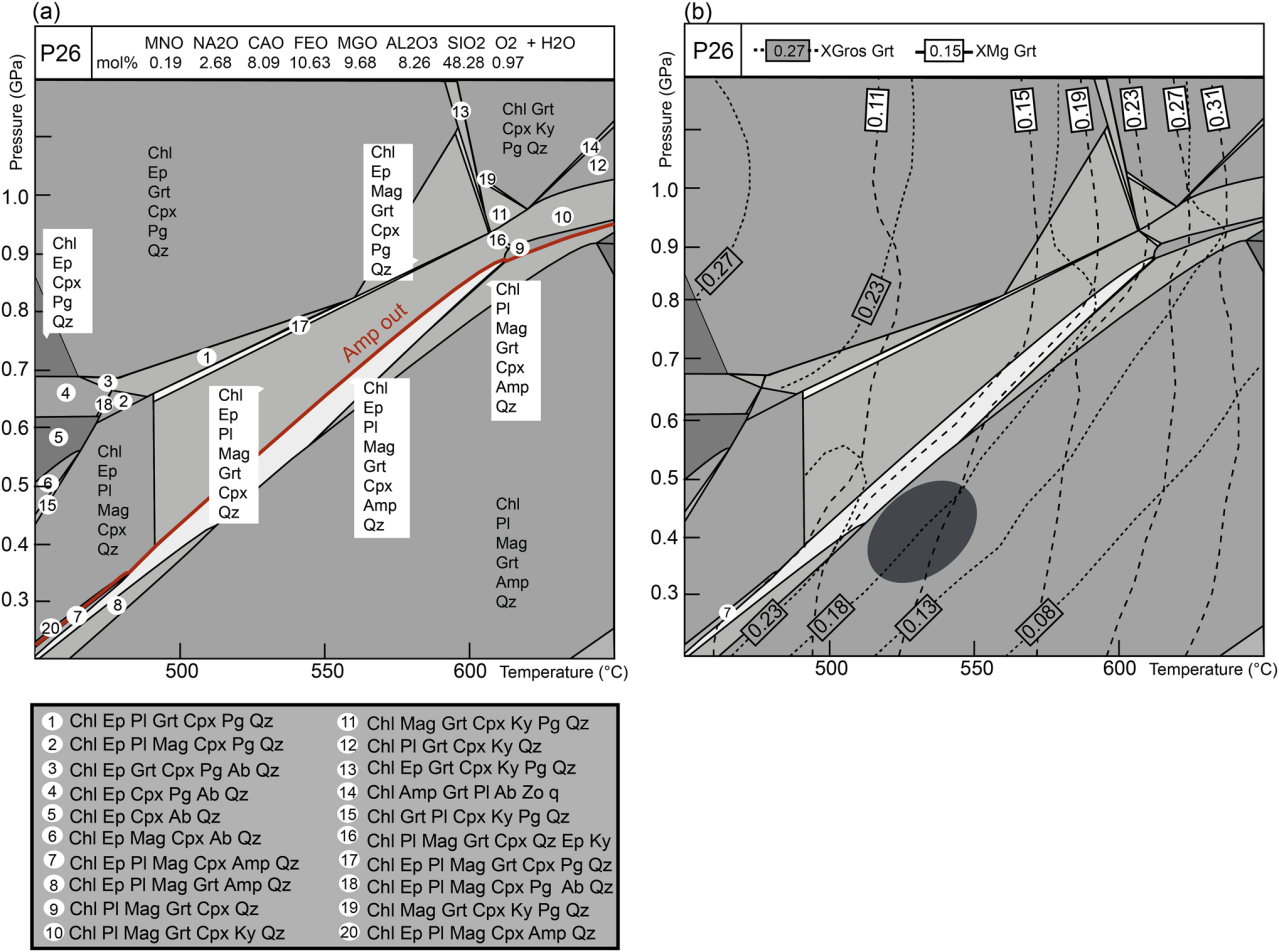
Phase diagram section and compositional isopleth of sample P26. **a** Phase diagram section calculated in the MnNCFMASHO system with H_2_O in excess. The red line indicates the boundary between clinopyroxene and amphibole stability fields. **b** Isopleths of *X*_Mg_ and *X*_Gros_ in garnet. The dark ellipse represents the inferred *P–T* conditions

The bulk-rock compositions (in mol%) used for the calculations are indicated in the upper part of the calculated *P–T* diagrams (Figs. 10 and 11). The bulk composition was obtained by X-ray fluorescence (XRF; Online Resource Table S.1). To estimate the appropriate amount of the O​_2_ component in the calculation for sample P26, a T-X_O2_ diagram was calculated (Online Resource Fig. S1).

### Mafic sample P4

The phase equilibria modelling shows the occurrence of zoisite in the high pressure-low temperature part of the *P–T* diagram (Fig. 10a). The occurrence of zoisite is accompanied by a drop in the *X*_An_ in plagioclase, where the low *X*_An_ values correspond to the high pressure-low temperature corner of the diagram, and the high X_An_ values to the low pressure-high temperature corner (Fig. 10b). Furthermore, the phase diagram section of sample P4 shows that amphibole is stable in the entire diagram (Fig. 10a).

Comparing the petrographic observations with the calculated results, albitic plagioclase grains in the matrix most likely represent relicts of the primary high-pressure mineralogy. The high *X*_An_ values (oligoclase compositions) in plagioclase intergrown with chlorite in the matrix and replacing garnet fits the lower pressure part of the diagram. Comparing the garnet chemical profile with the calculated diagram, the peak and post-pressure peak metamorphic path can be inferred. The peak pressure conditions are estimated using the *X*_Mg_ in garnet core (Table 4) and the albitic plagioclase relics. This corresponds to the stability field in the upper left corner of the diagram: Amp–Grt–Zo–Pg–Ab–Qz field around 510 °C and 1.00 GPa. The contours of *X*_Mg_ of the garnet rim and *X*_An_ of oligoclase in the matrix (Table 4) cross in the stability field: Chl–Amp–Pl–Grt–Qz (Fig. 10b). The *P–T* conditions of the lower pressure overprint were estimated at about 580 °C and 0.7 GPa.

**Table 4:** The *X*_Mg_ in garnet core and rim and the *X*_An_ in plagioclase from the mafic rocks

Sample	*X*_Mg_ garnet core	*X*_Mg_ garnet rim	*X*_An_ plagioclase
P4	0.05–0.08	0.12–0.14	0.19 (Oli) 0.01–0.06 (Ab)
P13	0.05–0.07	0.12–0.16	0.27–0.38 (Oli) 0.02–0.04 (Ab)
P26	0.07–0.08	0.10–0.13	0.04 (Ab)

The *X*_An_ corresponds to two endmembers: oligoclase (Oli) and albite (Ab)

### Mafic sample P13

The calculated phase diagram section based on the bulk rock composition shows that amphibole is stable everywhere except in the HP/LT region of the diagram (Fig. 10c). Furthermore, in the low-temperature–high-pressure region of the *P–T* diagram, zoisite is present and is accompanied by a drop in the *X*_An_ value in plagioclase (Fig. 10d). The albitic plagioclase observed in the sample thus most likely represents the relics of the primary mineralogy. The chemically zoned garnet can be used to infer the *P–T* path. The *X*_Mg_ value of the garnet core (Table 4) together with the observation of the albitic relics corresponds to the region at around 490 °C and 1.0 GPa in the Amp–Grt–Zo–Pg–Ab–Qz field (Fig. 10d). As indicated earlier, the composition of the oligoclase-rich plagioclase present in the matrix corresponds to the lower pressure part of the diagram (Fig. 10d). Due to the elliptical character of the garnet with patchy regions at the elongated parts (Fig. 6b), the representative *X*_Mg_ value of the garnet rim was taken from the contact with the matrix perpendicular to the profile measured along the elongated direction in Fig. 7b. Such *X*_Mg_ value corresponds to about 10 µm from the garnet rim (Fig. 7b, Table 4). The field with the observed mineral assemblage in the matrix (Chl–Amp–Pl–Grt–Qz) spreads across a larger part of the *P–T* diagram at low pressures, 0.3–0.7 GPa and 500–600 °C. The contours for *X*_Mg_ of the garnet rim and *X*_An_ in plagioclase (Table 4) correspond to the *P–T* conditions of about 550–600 °C and 0.5–0.6 GPa.

### Mafic sample P26

The calculated phase diagram section shows a clear distinction between amphibole-bearing high temperature-low pressure corner and clinopyroxene-bearing low temperature and high-pressure corner (Fig. 11a). The observed mineral assemblage corresponds to the large field Chl–Pl–Mag–Grt–Amp–Qz in the lower right corner. The contours of the garnet core *X*_Mg_ do not appear on the diagram, which prevents more detailed estimates of the *P–T* path. In the absence of oligoclase-rich plagioclase, the *X*_Mg_ of the garnet rim had to be compared against the slightly more pressure-dependent *X*_Gros_ of garnet (Tables 2, 4) to infer the *P–T* conditions of the matrix re-equilibration at about 530 °C and 0.4 GPa (Fig. 11b).

## Geochronology—zircon U–Pb dating

### Mafic rocks

**Fig. 12:**
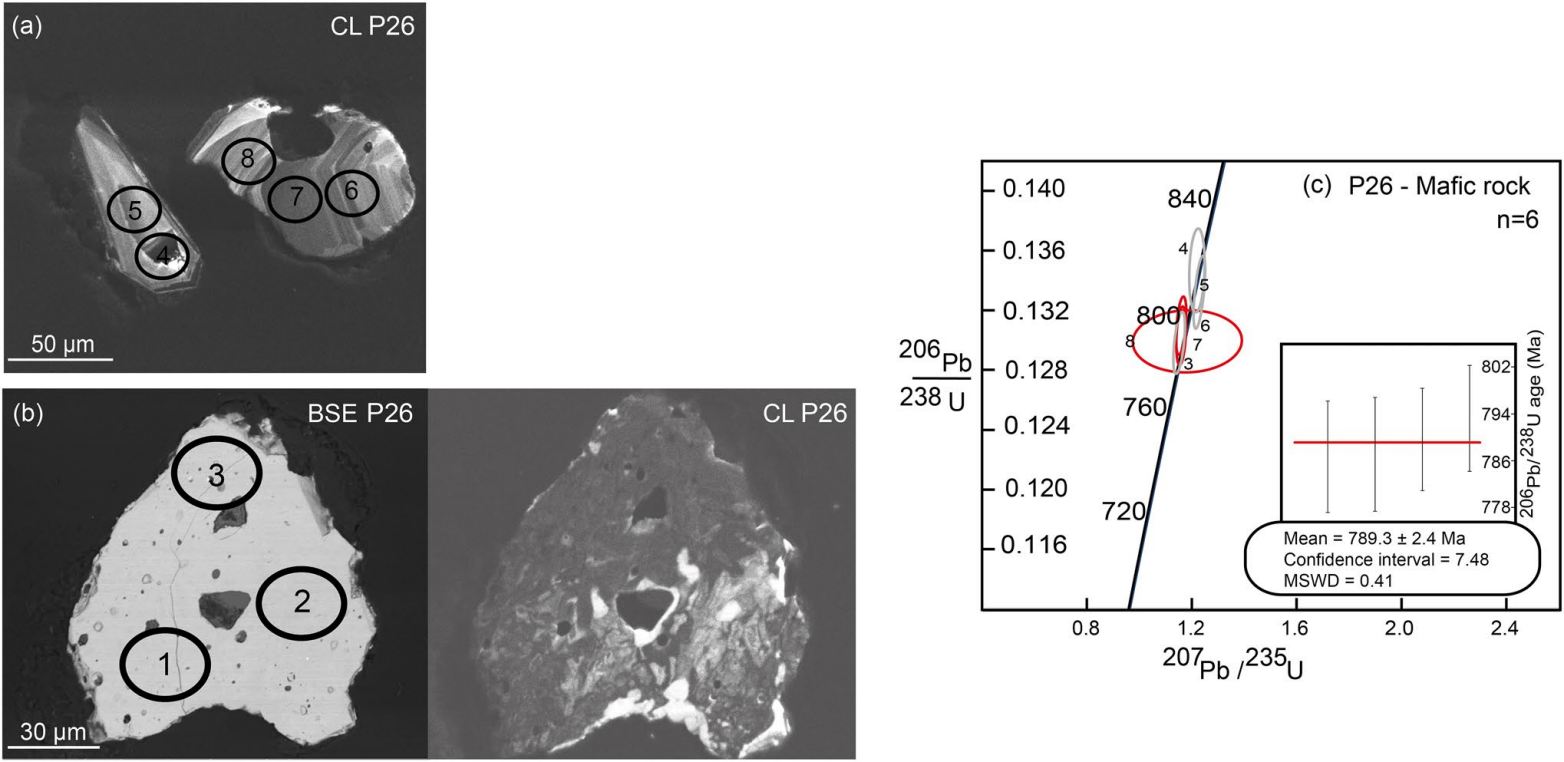
Zircon morphology and U–Pb dating from sample P26. **a**, **b** CL and BSE images of zircons with LA-ICPMS measurements indicated. **c** Concordia diagram shows concordant measurements. Measurement 4 and 5 give an age of 815–805 Ma and the main cluster of measurements (red circles: nr. 3,6,7,8) yield a mean age of 789.3 ± 2.4 Ma

Only three zircon grains were recovered from the mineral separation of sample P26, most probably due to the low total amount of zirconium in this rock. The zircons have a subhedral to anhedral shape. They are light pinkish in colour and transparent, 80–100 µm long with a width/length ratio of 1:1 to 1:2. In cathodoluminescence, two grains display growth zoning (Fig. 12a). Most of the U–Pb analyses plot on the Concordia. Analyses on the zoned zircon crystals with spot analyses 6, 7, and 8 show similar ages for core and rim of ca. 790 Ma. The analyses 4 and 5 in the zoned zircon are also concordant but yield slightly older ages, and display core to rim systematics from 815 to 805 Ma (Online Resource Table S.3). The third grain has a dark cathodoluminescence (CL) emission and shows no distinct regular zoning, but a mottled pattern, and it contains numerous pores and inclusions (Fig. 12b). For this grain, only spot number 3, with an age of 787 Ma, was used in the mean age calculation. The other two analyses in this grain (res. 1 and 2) are either not concordant or show a very large uncertainty. Therefore, these analyses were not considered for age calculation. The main cluster of measurements for this sample (res. 3, 6, 7, and 8) yield a mean age of 789.3 ± 2.4 Ma, with a Mean Square of the Weighted Deviates (MSWD) of 0.41 and a confidence interval of the weighted mean age of 7.48 (Fig. 12c). We set the average age for this sample between 815 and 790 Ma.

### Granodiorite

**Fig. 13:**
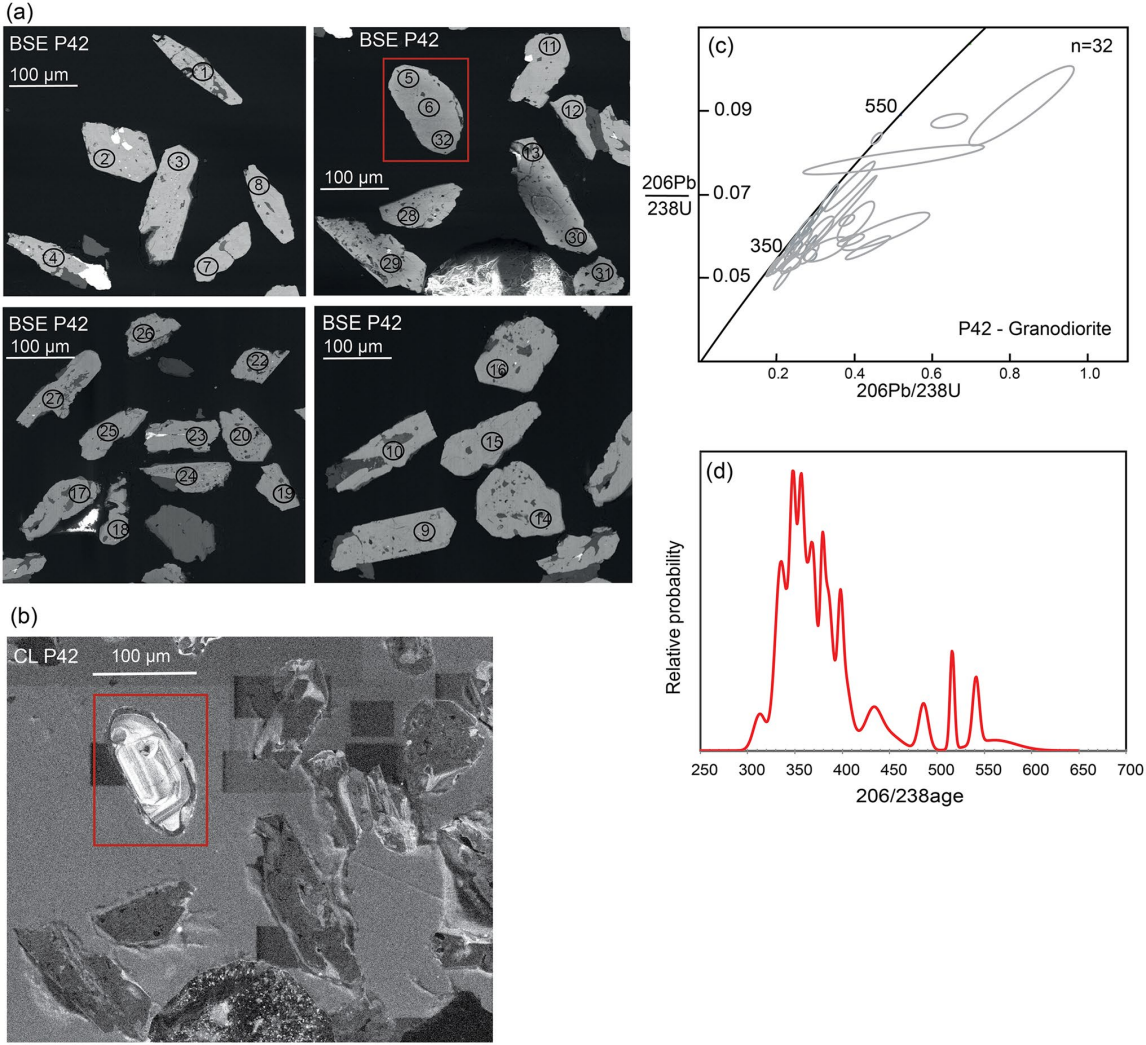
Zircon morphology and U–Pb dating from sample P42. **a** BSE images of zircons with LA-ICPMS measurements. **b** CL image of upper right BSE image in (**a**) showing only zonation in one zircon grain (within the red rectangle). **c** Concordia diagram showing wide spread of ages. **d** Probability diagram indicates age interval of 330–370 Ma

Zircon grains from granodiorite sample P42 have a subhedral to euhedral and mostly prismatic shape, 70–180 µm long with a width/length ratio of 1:3 (Fig. 13a). They have a milky colour and display no zoning, except for one grain (Fig. 13b). They contain numerous inclusions. The absence of zoning could be due to alteration (Rubatto and Hermann 2007). Most of the U–Pb analyses are discordant and show high U values (Online Resource Table S.3, Fig. 13c). The calculated ages have a large uncertainty and scatter from 300 to 500 Ma. The probability diagram (based on the 206/238 age of 32 measurements) shows a concentration of ages around 330–370 Ma (Fig. 13d).

## Discussion

### Origin of zircons in mafic rocks

Dating of mafic dykes using zircons is not straightforward (e.g., Black et al. (1991); García et al. (2010)), because inherited components may be incorporated into continental basalts by either shallow contamination or deep mixing. Black et al. (1991) and García et al. (2010) found zoned zircons in mafic dykes and concluded that some were inherited from the host rock. Xu et al. (2018) suggested that detrital zircons can be carried by terrigenous sediments into a subcontinental subduction zone, where the zircon can be transferred by fluids into the magma sources of continental basalts. Common to these studies is the relatively large number of zircons found in one single dyke, and the large spread in age between inherited and non-inherited zircons: in Black et al. (1991) and García et al. (2010) the inherited ages correspond to the ages of the host rock, where zircon ages vary widely from 1025 to 2483 Ma in the former, and from 310 to 2773 Ma in the latter; in Xu et al. (2018) the inherited ages vary widely from 101 to 3015 Ma. These two main characteristics of inherited zircon were not found in the studied mafic dyke. What further evidence do we have to support the igneous, non-inherited nature of the three zircons we found in our samples? (1) Two of the three zircons show the typical magmatic zoning (e.g., Corfu et al. (2003)). The third zircon with a dark cathodoluminescence emission shows no distinct regular zoning, which indicates a high degree of metamictization (internal radiation damage that leads to degradation of the minerals crystal structure) and could justify the absence of oscillatory zoning. (2) The host rock in our study is marble, a rock type in which unzoned zircons are the rule (Cavosie et al. 2011). Therefore, the analysed zircons with oscillatory zoning are not expected to be inherited from the host marble. (3) The obtained ages are restricted to the interval 787 to 815 Ma (28 Ma spread), which is roughly the same age within uncertainty, in contrast to the 48 Ma spread in García et al. (2010) (481 to 529 Ma), and the 211 Ma spread in Black et al. (1991) (864 to 1075 Ma). (4) The Zr-content in the studied rocks is low (ca. 88 ppm), as expected for a mafic rock. This could explain the scarcity of zircon crystals. (5) The U-content in the studied dyke varies between 120 and 425 ppm, with average around 239 ppm, which is similar to the average U-content in the magmatic zircons of García et al. (2010) (U-content average around 227 ppm), and much lower than the U-content in magmatic zircons of Black et al. (1991) (spread between 350 and 1720 ppm). The low Zr and U contents in the studied rocks are not typical of contamination by continental crust. (6) Last but not least, the analyzed zircon grains do not show any evidence of corrosion due to incorporation in a mafic magma at > 1200 ºC. To conclude, we did not find any evidence for inherited zircons in the studied mafic dyke.

If the analyzed zircons were inherited, then the age of the dyke would be younger. However, the age of the dyke cannot be much younger, because: (1) the studied mafic dykes intrude marbles, but not the Série Negra hosting the marbles; (2) the dykes and marbles record blueschist facies, which is not recorded in the Série Negra; (3) from 1 and 2, we infer that the marbles were not together with Série Negra at the time of dyke intrusion and HP/LT event, and that the marbles and enclosed dykes share a complex evolution (from an initial sedimentary stage to subduction and later exhumation—a full orogenic cycle) that cannot have happened in just a few millions of years; (4) the age of the Série Negra has been set at about 550 Ma; therefore, the dykes and host marble must be significantly older, most likely the time of a full orogenic cycle and initiation of a new one. Moreover, from (1) and (2) we further infer that the HP-LT bodies in Série Negra are pieces of an older lithosphere later incorporated in the Ediacaran sediments, likely as olistoliths, because their size varies from meters to hectometers. Moreover, we did not observe tectonic contacts between HP-LT bodies and the Ediacaran sediments.

### Age of the Série Negra in the study area

We present a new age of ca. 815–790 Ma obtained by U–Pb dating on zircon from the mafic rocks intruded in marbles found in the Série Negra. The new age is interpreted as a magmatic age given the typical magmatic zoning in zircon (e.g., Corfu et al. (2003)) and discussion above. As age of the protolith, it sets an upper limit for the age of HP-LT metamorphism in the studied rocks. This new age cannot help clarifying the age of Série Negra, because: (1) two kinds of marbles have been mapped, one below the Série Negra and the other in the Série Negra; (2) the relationship between the dated marbles with HP-LT mineral associations and the surrounding Série Negra is unknown. According to the current knowledge, the Série Negra in the study area has not been affected by HP-LT metamorphism. Therefore, the marbles with HP-LT mafic rocks cannot be directly correlated with the host Série Negra. They are interpreted as olistoliths.

### *P–T* evolution and timing

The metamorphic evolution shows two stages: (1) HP/LT event at about 1.0 ± 0.1 GPa and 470–510 °C, indicated by relics of glaucophane grains, and (2) MP/MT overprint at about 0.6 ± 0.2 GPa and 550–600 °C. Since we did not date the metamorphic events, we can only discuss the most likely age of peak metamorphism. Moita et al. (2005) used Sm/Nd on garnet, and ^40^Ar/^39^Ar on amphibole of retrogressed rocks to date the HP/L-MT metamorphism at ca. 370 Ma, coincident, within error, with the oldest age on amphibole. This implies that peak metamorphism and retrogression were very close, or that the Sm/Nd age has been reset by the thermal episode at ca. 360 Ma. Rosas et al. (2008) argued for an age > 360 Ma for the HP-LT metamorphism, and interpreted the ca. 360 Ma age as close to the metamorphic peak of the event that affected the metasedimentary rocks. Therefore, they concluded that the age of the sedimentary protolith of the Série Negra is older. The new data reported in the present work suggests a different interpretation. Given that the Ediacaran sediments of Série Negra are ca. 550 Ma old, and that the HP-LT olistholiths in the Série Negra have a different metamorphic history, the blueschist metamorphism should be older than 550 Ma, i.e., Cadomian (between ca. 790 and 550 Ma). From the oldest U–Pb ages of ca. 355 Ma on zircons from the Beja Igneous Complex, which are, within error, undistinguishable from the muscovite age in the Série Negra of the study area (ca. 360 Ma), Rosas et al. (2008) concluded that the gabbro intrusion is responsible for the muscovite growth and related deformation in Série Negra. The 360 Ma age may date cooling after heating around the Beja Igneous Complex. The 330–370 Ma age of the granodioritic dyke dated in this study can either be related to the intrusion of the Beja Igneous Complex and/or to the intrusion of the granodiorites of the Évora Massif, dated 340–317 Ma (Lima et al. 2012; Moita et al. 2015; Pereira et al. 2009, 2015; Rosas et al. 2008). The rise in metamorphic temperature between the two metamorphic stages is here related to the emplacement of either of these magmatic bodies.

### Correlation between the OMZ and the Iberian Autochthon

Previous works summarized in Sánchez-García et al. (2016) have shown ca. 620 Ma zircon xenocrysts in volcanic rocks. Those were interpreted as the source for Late Ediacaran sediments. Sánchez-García et al. (2016) and others (e.g., Álvaro et al. (2014); Henriques et al. (2015); Henriques et al. (2017); Linnemann et al. (2008); Sánchez-Lorda et al. (2016)) tentatively correlated the OMZ with the North Armorican Zone in Brittany, Normandy and the Channel Islands, the Neoproterozoic of the Saxo-Thuringian and Teplá-Barrandian zones in the Bohemian Massif, and the arc systems currently exposed in the Moroccan Anti-Atlas.

The 815–790 Ma intrusion age of the HP-LT rocks in marbles in the Série Negra has important consequences regarding the correlation of the OMZ with the neighboring terranes. This age compels an older age of the host rocks, and falls in the age gap between Neoproterozoic (ca. 750–540 Ma) and Paleoproterozoic (ca. 2.0 Ga) found in zircon provenance analyses in the OMZ (e.g., Fernández-Suárez et al. (2002); López-Guijarro et al. (2007); Pereira et al. (2011); Pereira et al. (2008); Pereira et al. (2012)). The new age also falls in the ca. 850–550 Ma interval found by provenance studies in Late Ediacaran—Early Paleozoic sequences in the Iberian Massif (e.g., Fernández-Suárez et al. (2002); Gutiérrez-Alonso et al. (2005); Gutiérrez-Alonso et al. (2015)), thus indicating an affinity between the Ossa-Morena and Central Iberian zones.

### Geodynamic setting

**Fig. 14:**
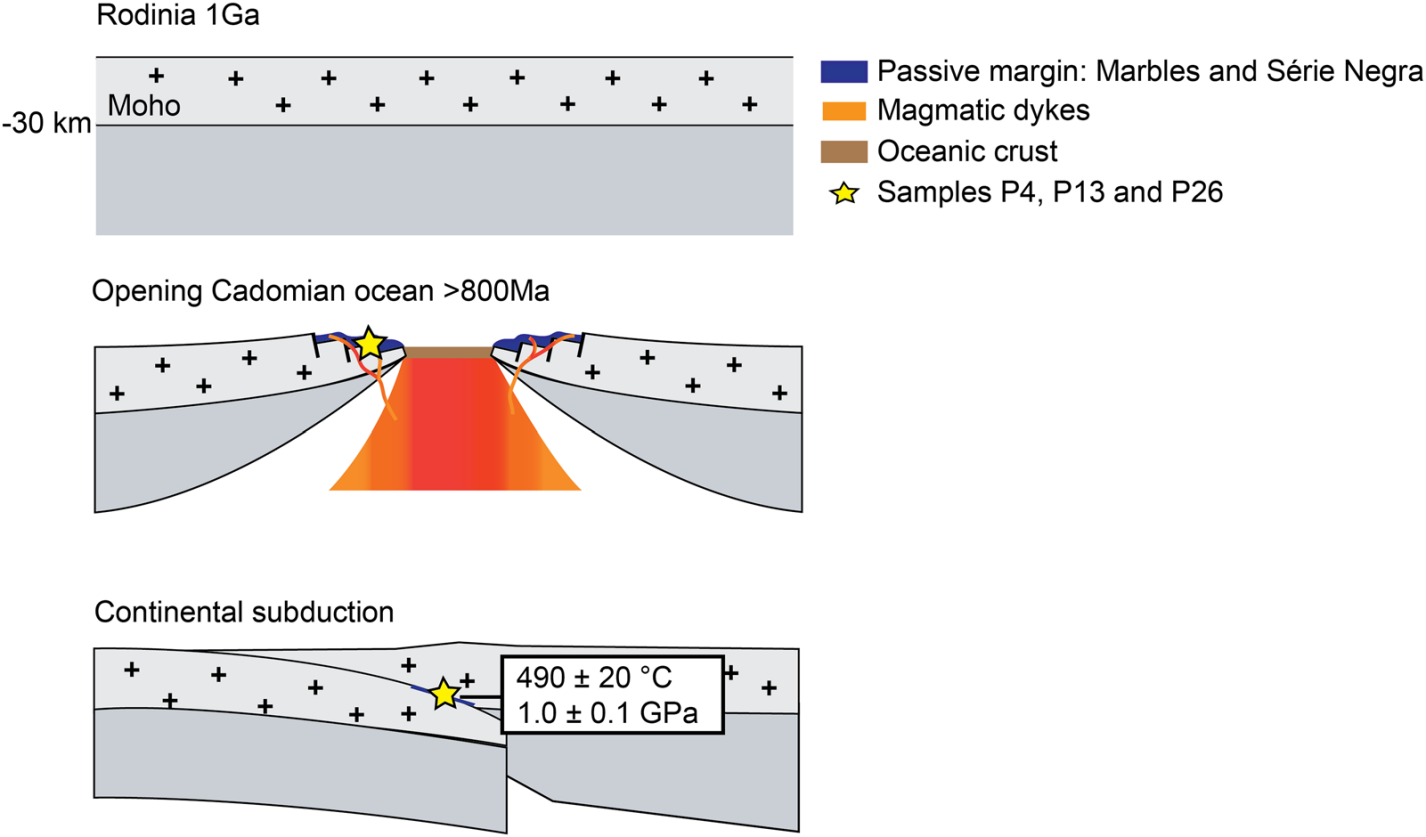
Late Proterozoic evolution, from Grenvillian Rodinia (ca. 1 Ga), to the opening of a Cadomian ocean (> ca. 800 Ma – marbles and Série Negra on a passive continental margin), and continental subduction with marbles and Série Negra being carried down to blueschist/eclogite conditions. For illustrative reasons, we estimate depth from lithostatic pressure: *z* = *P*/ρg = 0.7 GPa/(2700 kgm^−3^ × 10 ms^−2^) = ca. –27 km

The geodynamic evolution of the OMZ comprises the Cadomian cycle, with rapid transition from a Cadomian subduction-related regime (onset of Cadomian arc magmatism dated at ca. 692 Ma by Henriques et al. (2015) and ca. 645 Ma by Sánchez-Lorda et al. (2016)) to intracontinental rifting in the Cambrian, which corresponds to the onset of the Variscan Cycle. The new U–Pb zircon crystallization age of 815–790 Ma for mafic rocks in marbles in the Série Negra suggests an untold story before Cadomian subduction (Fig. 14). At this stage, a passive margin formed, where limestones (now the marbles) were deposited and intruded by magmatic dykes (now the mafic rocks). Later, these rocks experienced undated HP-LT metamorphism. This metamorphism has been related to either the Cadomian or the Variscan subduction. The relation between blueschist facies basic olistoliths in the Série Negra, and the age of Série Negra (ca. 550 Ma), preclude the possibility that the studied HP-LT metamorphism is Variscan (i.e., Early to Late Paleozoic).

## Conclusion

In this study, the protolith of HP-LT mafic rocks scattered in Ediacaran metasediments, (so-called Série Negra) of the southwestern OMZ, was dated at about 815–790 Ma. Having considered all the possible uncertainties related to the dating of mafic rocks, this age is taken as the crystallization age of the tholeiitic magma. If so, the new age is the oldest in the OMZ and constrains the minimum age of the pre-Cadomian basement, because the intruded sediments must be older. The mafic rocks may represent magmatic dykes in a continental shelf, and may be correlated to the opening of a Cadomian Ocean before 815 Ma. The similarity between the new age and the 850–550 Ma age interval found by provenance studies in Late Ediacaran—Early Palaeozoic sequences in the Iberian Massif indicates an affinity between the Ossa-Morena and Central Iberian zones. Furthermore, *P–T* conditions of two metamorphic events in the mafic rocks were inferred from phase equilibria modelling: (1) a HP/LT event of ca. 1.0 ± 0.1 GPa and 470–510 °C, and (2) a MP/HT event at ca. 0.6 ± 0.2 GPa and 550–600 °C. The age of peak metamorphism is unspecified, but the increase in metamorphic temperature between the two stages is attributed to the intrusion of the Beja Igneous Complex and/or the Évora Massif. Given that Série Negra (ca. 550 Ma old) and the studied mafic rocks do not have the same metamorphic history, the blueschist episode is Cadomian (between ca. 790 and 550 Ma).

## Electronic supplementary material

Below is the link to the electronic supplementary material.

Supplementary file1 (PDF 350 kb)

Supplementary file2 (XLSX 30 kb)

## Data Availability

The main data are provided and the additional upon request to VA or LT.

## References

[CR1] Abalos B, Ibarguchi JG, Eguiluz L (1991). Cadomian subduction/collision and Variscan transpression in the Badajoz-Córdoba shear belt, southwest Spain. Tectonophysics.

[CR2] Álvaro JJ, Bellido F, Gasquet D, Pereira MF, Quesada C, Sánchez-García T (2014). Diachronism in the late Neoproterozoic-Cambrian arc-rift transition of North Gondwana: a comparison of Morocco and the Iberian Ossa-Morena Zone. J Afr Earth Sci.

[CR3] Andersen DJ, Lindsley DH (1988). Internally consistent solution models for Fe–Mg–Mn–Ti oxides: Fe–Ti oxides. Am Mineral.

[CR4] Araújo A, Fonseca P, Munhá J, Moita P, Pedro J, Ribeiro A (2005). The Moura Phyllonitic Complex: an accretionary complex related with obduction in the Southern Iberia Variscan Suture. Geodin Acta.

[CR5] Azor A, Simancas J, Poyatos DM, Montero P, Lodeiro FG, Pérez-Cáceres I (2016) U–Pb zircon age and tectonic meaning of the Cardenchosa pluton (Ossa-Morena Zone). In :IX Congreso Geológico de España, Geo-Temas, vol 16, no. 2, pp. 23–26

[CR6] Black LP, Kinny PD, Sheraton JW (1991). The difficulties of dating mafic dykes: an Antarctic example. Contrib Mineral Petr.

[CR7] Black LP, Kamo SL, Allen CM, Aleinikoff JN, Davis DW, Korsch RJ, Foudoulis C (2003). TEMORA 1: a new zircon standard for Phanerozoic U-Pb geochronology. Chem Geol.

[CR8] Casado BO (1998) Geochronological studies of the Pre-Mesozoic basement of the Iberian Massif: the Ossa Morena zone and the Allochthonous Complexes within the Central Iberian zone, doctoral dissertation, ETH Zurich

[CR9] Cavosie AJ, Valley JW, Kita NT, Spicuzza MJ, Ushikubo T, Wilde SA (2011). The origin of high δ 18 O zircons: marbles, megacrysts, and metamorphism. Contrib Mineral Petr.

[CR10] Chichorro M, Pereira M, Diaz-Azpiroz M, Williams I, Fernández C, Pin C, Silva JB (2008). Cambrian ensialic rift-related magmatism in the Ossa-Morena Zone (Évora–Aracena metamorphic belt, SW Iberian Massif): Sm–Nd isotopes and SHRIMP zircon U–Th–Pb geochronology. Tectonophysics.

[CR11] Connolly J (2009) The geodynamic equation of state: what and how. Geochemistry, Geophysics, Geosystems 10

[CR12] Corfu F, Hanchar JM, Hoskin PW, Kinny P (2003). Atlas of zircon textures. Rev Mineral Geochem.

[CR13] Crespo-Blanc A, Orozco M (1988). The Southern Iberian Shear Zone: a major boundary in the Hercynian folded belt. Tectonophysics.

[CR14] da Silva ÍD, Pereira MF, Silva JB, Gama C (2018). Time-space distribution of silicic plutonism in a gneiss dome of the Iberian Variscan Belt: The Évora Massif (Ossa-Morena Zone, Portugal). Tectonophysics.

[CR15] Dale J, Powell R, White R, Elmer F, Holland T (2005). A thermodynamic model for Ca–Na clinoamphiboles in Na2O–CaO–FeO–MgO–Al2O3–SiO2–H2O–O for petrological calculations. J Metamoph Geol.

[CR16] Dallmeyer R, Quesada C (1992). Cadomian vs. Variscan evolution of the Ossa-Morena Zone (SW Iberia): field and 40Ar/39Ar mineral age constraints. Tectonophysics.

[CR17] Dallmeyer R, Fonseca P, Quesada C, Ribeiro A (1993). 40Ar/39Ar mineral age constraints for the tectonothermal evolution of a Variscan suture in southwest Iberia. Tectonophysics.

[CR18] de Oliveira JT, Oliveira V, Piçarra JM (1991) Traços gerais da evolução tectono-estratigráfica da Zona de Ossa-Morena, em Portugal

[CR19] Eguiluz L (1987) Petrogénesis de rocas ígneas y metamórficas en el antiforme Burguillos-Monesterio. Macizo Ibérico Meridional PhD, Univ País Vasco, 456p

[CR20] Eguiluz L, Ibarguchi JG, Abalos B, Apraiz A (2000). Superposed Hercynian and Cadomian orogenic cycles in the Ossa-Morena zone and related areas of the Iberian Massif. Geol Soc Am Bull.

[CR21] Fernández-Suárez J, Alonso GG, Jeffries T (2002). The importance of along-margin terrane transport in northern Gondwana: insights from detrital zircon parentage in Neoproterozoic rocks from Iberia and Brittany. Earth Planet Sci Lett.

[CR22] Floyd P, Winchester J (1975). Magma type and tectonic setting discrimination using immobile elements. Earth Planet Sci Lett.

[CR23] García FD, Martínez SS, Castiñeiras P, Fuenlabrada J, Arenas R (2010). A peri-Gondwanan arc in NW Iberia. II: assessment of the intra-arc tectonothermal evolution through U–Pb SHRIMP dating of mafic dykes. Gondwana Res.

[CR24] Gómez-Pugnaire M, Azor A, Fernández-Soler J, Sánchez-Vizcaıno VL (2003). The amphibolites from the Ossa–Morena/Central Iberian Variscan suture (Southwestern Iberian Massif): geochemistry and tectonic interpretation. Lithos.

[CR25] Gutiérrez-Alonso G, Fernández-Suárez J, Collins AS, Abad I, Nieto F (2005). Amazonian Mesoproterozoic basement in the core of the Ibero-Armorican Arc: 40Ar/39Ar detrital mica ages complement the zircon's tale. Geology.

[CR26] Gutiérrez-Alonso G (2015). Significance of detrital zircons in Siluro-Devonian rocks from Iberia. J Geol Soc London.

[CR27] Henriques S, Neiva A, Ribeiro M, Dunning G, Tajčmanová L (2015). Evolution of a Neoproterozoic suture in the Iberian Massif, Central Portugal: new U–Pb ages of igneous and metamorphic events at the contact between the Ossa Morena Zone and Central Iberian Zone. Lithos.

[CR28] Henriques S, Neiva A, Tajčmanová L, Dunning G (2017). Cadomian magmatism and metamorphism at the Ossa Morena/Central Iberian zone boundary, Iberian Massif, Central Portugal: geochemistry and P–T constraints of the Sardoal Complex. Lithos.

[CR29] Holland T, Powell R (1996). Thermodynamics of order-disorder in minerals: II. Symmetric formalism applied to solid solutions. Am Mineral.

[CR30] Holland T, Powell R (1998). An internally consistent thermodynamic data set for phases of petrological interest. J Metamorph Geol.

[CR31] Jackson SE, Pearson NJ, Griffin WL, Belousova EA (2004). The application of laser ablation-inductively coupled plasma-mass spectrometry to in situ U–Pb zircon geochronology. Chem Geol.

[CR32] Jesus A, Munhá J, Mateus A, Tassinari C, Nutman AP (2007). The Beja layered gabbroic sequence (Ossa-Morena Zone, Southern Portugal): geochronology and geodynamic implications. Geodin Acta.

[CR33] Jesus AP, Mateus A, Munhá JM, Tassinari CC, dos Santos TMB, Benoit M (2016). Evidence for underplating in the genesis of the Variscan synorogenic Beja Layered Gabbroic Sequence (Portugal) and related mesocratic rocks. Tectonophysics.

[CR34] Leake BE (1997). Report. Nomenclature of amphiboles: report of the subcommittee on amphiboles of the international mineralogical association commission on new minerals and mineral names. Mineral Mag.

[CR35] Li Z-X (2008). Assembly, configuration, and break-up history of Rodinia: a synthesis. Precambrian Res.

[CR36] Lima S, Corfu F, Neiva AMR, Ramos J (2012). Dissecting complex magmatic processes: an in-depth U–Pb study of the Pavia pluton, Ossa-Morena Zone, Portugal. J Petrol.

[CR37] Linnemann U, Gerdes A, Drost K, Buschmann B (2007). The continuum between Cadomian orogenesis and opening of the Rheic Ocean: Constraints from LA–ICP–MS U–Pb zircon dating and analysis of plate-tectonic setting (Saxo-Thuringian zone, northeastern Bohemian Massif, Germany). Geol Soc Am S.

[CR38] Linnemann U, Pereira F, Jeffries TE, Drost K, Gerdes A (2008). The Cadomian Orogeny and the opening of the Rheic Ocean: the diacrony of geotectonic processes constrained by LA-ICP-MS U-Pb zircon dating (Ossa-Morena and Saxo-Thuringian Zones, Iberian and Bohemian Massifs). Tectonophysics.

[CR39] López-Guijarro R, Quesada C, Fernández-Suárez J, Jeffries T, Pin C (2007) Age of the rift–drift transition of the Rheic Ocean in the Ossa-Morena zone: K-bentonite in the Early Ordovician succession at “Venta del Ciervo”. In: The rootless Variscan suture of NW Iberia (Galicia, Spain). Abstracts and Programme IGCP-497 Meeting, The Rheic Ocean: Its origin, evolution and correlatives, 2007. Publicaciones del Instituto Geológico y Minero de España, Madrid, pp 142–143

[CR40] López-Guijarro R, Armendáriz M, Quesada C, Fernández-Suárez J, Murphy JB, Pin C, Bellido F (2008). Ediacaran-Palaeozoic tectonic evolution of the Ossa Morena and Central Iberian zones (SW Iberia) as revealed by Sm–Nd isotope systematics. Tectonophysics.

[CR41] Ludwig KR (2003) User's manual for IsoPlot 3.0 A geochronological toolkit for Microsoft Excel 71

[CR42] Massonne H-J, Willner AP (2008). Phase relations and dehydration behaviour of psammopelite and mid-ocean ridge basalt at very-low-grade to low-grade metamorphic conditions. Eur J Mineral.

[CR43] Miyashiro A (1978). Nature of alkalic volcanic rock series. Contrib Mineral Petr.

[CR44] Moita P, Munhá J, Fonseca P, Pedro J, Araújo A, Tassinari C, Palacios T (2005) Phase equilibria and geochronology of Ossa-Morena eclogites

[CR45] Moita P, Santos JF, Pereira MF (2009). Layered granitoids: interaction between continental crust recycling processes and mantle-derived magmatism: examples from the Évora Massif (Ossa–Morena Zone, Southwest Iberia, Portugal). Lithos.

[CR46] Moita P, Santos JF, Pereira M, Costa M, Corfu F (2015). The quartz-dioritic Hospitais intrusion (SW Iberian Massif) and its mafic microgranular enclaves—evidence for mineral clustering. Lithos.

[CR47] Murphy JB (2006). Origin of the Rheic Ocean: rifting along a Neoproterozoic suture?. Geology.

[CR48] Murphy JB, Pisarevsky SA, Nance RD, Keppie JD (2004). Neoproterozoic—early Paleozoic evolution of peri-Gondwanan terranes: implications for Laurentia-Gondwana connections. Int J Earth Sci.

[CR49] Nance RD (2010). Evolution of the Rheic ocean. Gondwana Res.

[CR50] Newton R, Charlu T, Kleppa O (1980). Thermochemistry of the high structural state plagioclases. Geochim Cosmochim Ac.

[CR51] Ochsner A (1993) U-Pb geochronology of the Upper Proterozoic-Lower Paleozoic geodynamic evolution in the Ossa-Morena Zone (SW Iberia), Doctoral dissertation, ETH Zurich

[CR52] Oliveira J, Pereira E, Ramalho M, Antunes M, Monteiro J (1992) 5ª Edição da Carta Geológica de Portugal na escala de 1: 500 000 Serviços Geológicos de Portugal

[CR53] Paton C, Hellstrom J, Paul B, Woodhead J, Hergt J (2011). Iolite: Freeware for the visualisation and processing of mass spectrometric data. J Anal Atom Spectrom.

[CR54] Pereira M, Apraiz A, Chichorro M, Silva JB, Armstrong R (2010). Exhumation of high-pressure rocks in northern Gondwana during the Early Carboniferours (Coimbra–Cordoba shear zone, SW Iberian Massif): tectonothermal analysis and U–Th–Pb SHRIMP in-situ zircon geochronology. Gondwana Res.

[CR55] Pereira MF, Chichorro M, Linnemann U, Eguiluz L, Silva JB (2006). Inherited arc signature in Ediacaran and Early Cambrian basins of the Ossa-Morena zone (Iberian Massif, Portugal): paleogeographic link with European and North African Cadomian correlatives. Precambrian Res.

[CR56] Pereira M, Chichorro M, Williams I, Silva JB (2008). Zircon U–Pb geochronology of paragneisses and biotite granites from the SW Iberian Massif (Portugal): evidence for a palaeogeographical link between the Ossa-Morena Ediacaran basins and the West African craton. Geol Soc Spec Publ.

[CR57] Pereira MF (2009). Variscan intra-orogenic extensional tectonics in the Ossa-Morena Zone (Évora-Aracena-Lora del Río metamorphic belt, SW Iberian Massif): SHRIMP zircon U–Th–Pb geochronology. Geol Soc Spec Publ.

[CR58] Pereira M, Chichorro M, Solá AR, Silva JB, Sánchez-García T, Bellido F (2011). Tracing the Cadomian magmatism with detrital/inherited zircon ages by in-situ U–Pb SHRIMP geochronology (Ossa-Morena Zone, SW Iberian Massif). Lithos.

[CR59] Pereira MF, Solá AR, Chichorro M, Lopes L, Gerdes A, Silva JB (2012). North-Gondwana assembly, break-up and paleogeography: U–Pb isotope evidence from detrital and igneous zircons of Ediacaran and Cambrian rocks of SW Iberia. Gondwana Res.

[CR60] Pereira M (2015). The multistage crystallization of zircon in calc-alkaline granitoids: U–Pb age constraints on the timing of Variscan tectonic activity in SW Iberia. Int J Earth Sci.

[CR61] Petrus JA, Kamber BS (2012). VizualAge: a novel approach to laser ablation ICP-MS U–Pb geochronology data reduction. Geostand Geoanal Res.

[CR62] Pin C, Fonseca PE, Paquette J-L, Castro P, Matte P (2008). The ca. 350 Ma Beja Igneous Complex: a record of transcurrent slab break-off in the Southern Iberia Variscan Belt?. Tectonophysics.

[CR63] Quesada C (1990). Precambrian successions in SW Iberia: their relationship to ‘Cadomian’orogenic events. Geol Soc Spec Publ.

[CR64] Quesada C (1991). Geological constraints on the Paleozoic tectonic evolution of tectonostratigraphic terranes in the Iberian Massif. Tectonophysics.

[CR65] Quesada C (1992) Evolución tectónica del Macizo Ibérico (Una historia de crecimiento por acrecencia sucesiva de terrenos durante el Proterozoico superior y el Paleozoico) Palaeozoico inferior de Ibero-América University of Extremadura, Mérida, 173–190

[CR66] Quesada C (2006). The Ossa-Morena Zone of the Iberian Massif: a tectonostratigraphic approach to its evolution. Z Dtsch Ges Geowiss.

[CR67] Quesada C, Dallmeyer R (1994). Tectonothermal evolution of the Badajoz-Córdoba shear zone (SW Iberia): characteristics and 40Ar/39Ar mineral age constraints. Tectonophysics.

[CR68] Quesada C (1991). Terranes within the Iberian Massif: correlations with West African sequences. The West African orogens and circum-Atlantic correlatives.

[CR69] Quesada C, Fonseca P, Munhá J, Oliveira J, Ribeiro A (1994). The Beja-Acebuches Ophiolite (Southern Iberia Variscan fold belt): geological characterization and geodynamic significance. Boletín Geológico y Minero.

[CR70] Ribeiro A, Quesada C, Dallmeyer R (1990). Geodynamic evolution of the Iberian Massif. Pre-Mesozoic geology of Iberia.

[CR71] Rosas FM (2003) Estudo tectónico do sector de Viana do Alentejo-Alvito: evolução geodinâmica e modelação analógica de estruturas em afloramentos chave (ramo Sul da Cadeia Varisca Ibérica-SW da zona de Ossa Morena).

[CR72] Rosas F, Marques F, Ballevre M, Tassinari C (2008). Geodynamic evolution of the SW Variscides: Orogenic collapse shown by new tectonometamorphic and isotopic data from western Ossa-Morena Zone, SW Iberia. Tectonics.

[CR73] Rubatto D, Jr H (2007) Zircon behaviour in deeply subducted rocks. Elements 3:31–35

[CR74] Sánchez-Garcıa T, Bellido F, Quesada C (2003). Geodynamic setting and geochemical signatures of Cambrian-Ordovician rift-related igneous rocks (Ossa-Morena Zone, SW Iberia). Tectonophysics.

[CR75] Sánchez-García T, Quesada C, Bellido F, Dunning G, Pin C, Moreno-Eiris E, Perejón A (2016). Age and characteristics of the Loma del Aire unit (SW Iberia): implications for the regional correlation of the Ossa-Morena Zone. Tectonophysics.

[CR76] Sánchez-Lorda M, Ábalos B, de Madinabeitia SG, Eguíluz L, Ibarguchi JG, Paquette J-L (2016). Radiometric discrimination of pre-Variscan amphibolites in the Ediacaran Serie Negra (Ossa-Morena Zone, SW Iberia). Tectonophysics.

[CR77] Sarrionandia F, Sánchez MC, Eguiluz L, Ábalos B, Rodríguez J, Pin C, Ibarguchi JG (2012). Cambrian rift-related magmatism in the Ossa-Morena Zone (Iberian Massif): geochemical and geophysical evidence of Gondwana break-up. Tectonophysics.

[CR78] Schäfer H-J, Gebauer D, Nägler TF, Eguiluz L (1993). Conventional and ion-microprobe U–Pb dating of detrital zircons of the Tentudia Group (Serie Negra, SW Spain): implications for zircon systematics, stratigraphy, tectonics and the Precambrian/Cambrian boundary. Contrib Mineral Petr.

[CR79] Simancas J, Poyatos DM, Expósito I, Azor A, Lodeiro FG (2001). The structure of a major suture zone in the SW Iberian Massif: the Ossa-Morena/Central Iberian contact. Tectonophysics.

[CR80] Sun S-S, McDonough W-s (1989). Chemical and isotopic systematics of oceanic basalts: implications for mantle composition and processes. Geol Soc Spec Publ.

[CR81] Whitney DL, Evans BW (2010). Abbreviations for names of rock-forming minerals. Am Mineral.

[CR82] Wiedenbeck M (1995). Three natural zircon standards for U-Th-Pb, Lu-Hf, trace element and REE analyses. Geostandard Newslett.

[CR83] Wilson M (1989). Igneous petrogenesis: a global tectonic approach.

[CR84] Xu Z, Zheng Y-F, Zhao Z-F (2018). Zircon evidence for incorporation of terrigenous sediments into the magma source of continental basalts. Sci Rep-UK.

