# Supplementary material for: U–Pb zircon geochronology and phase equilibria modelling of HP-LT rocks in the Ossa-Morena Zone, Portugal
Source: Int J Earth Sci. 2020 Sep 9;109(8):2719–38. doi: 10.1007/s00531-020-01921-w (PMC7581627; doi:10.1007/s00531-020-01921-w)
Supplement: Supplementary file 1 — Supplementary file1 (PDF 350 kb) [file 531_2020_1921_MOESM1_ESM.pdf]

International Journal of Earth Sciences

U-Pb zircon geochronology and phase equilibria modelling of HP-LT rocks in the Ossa-Morena Zone, Portugal

Ismay Vénice Akker[a], Lucie Tajčmanová[b], Fernando O. Marques[c], Jean-Pierre Burg[d]

[a] Institute of Geological Sciences, University of Bern, Baltzerstrasse 1+3, 3012 Bern, Switzerland

[b]Institute of Earth Science, Heidelberg University, Heidelberg, Germany

[c]Universidade de Lisboa, 1749-016 Lisboa, Portugal

[d] Earth Sciences Department, ETH Zürich, Sonneggstrasse 5, 8092 Zürich, Switzerland

Corresponding author: Lucie Tajčmanová (lucataj@gmail.com)

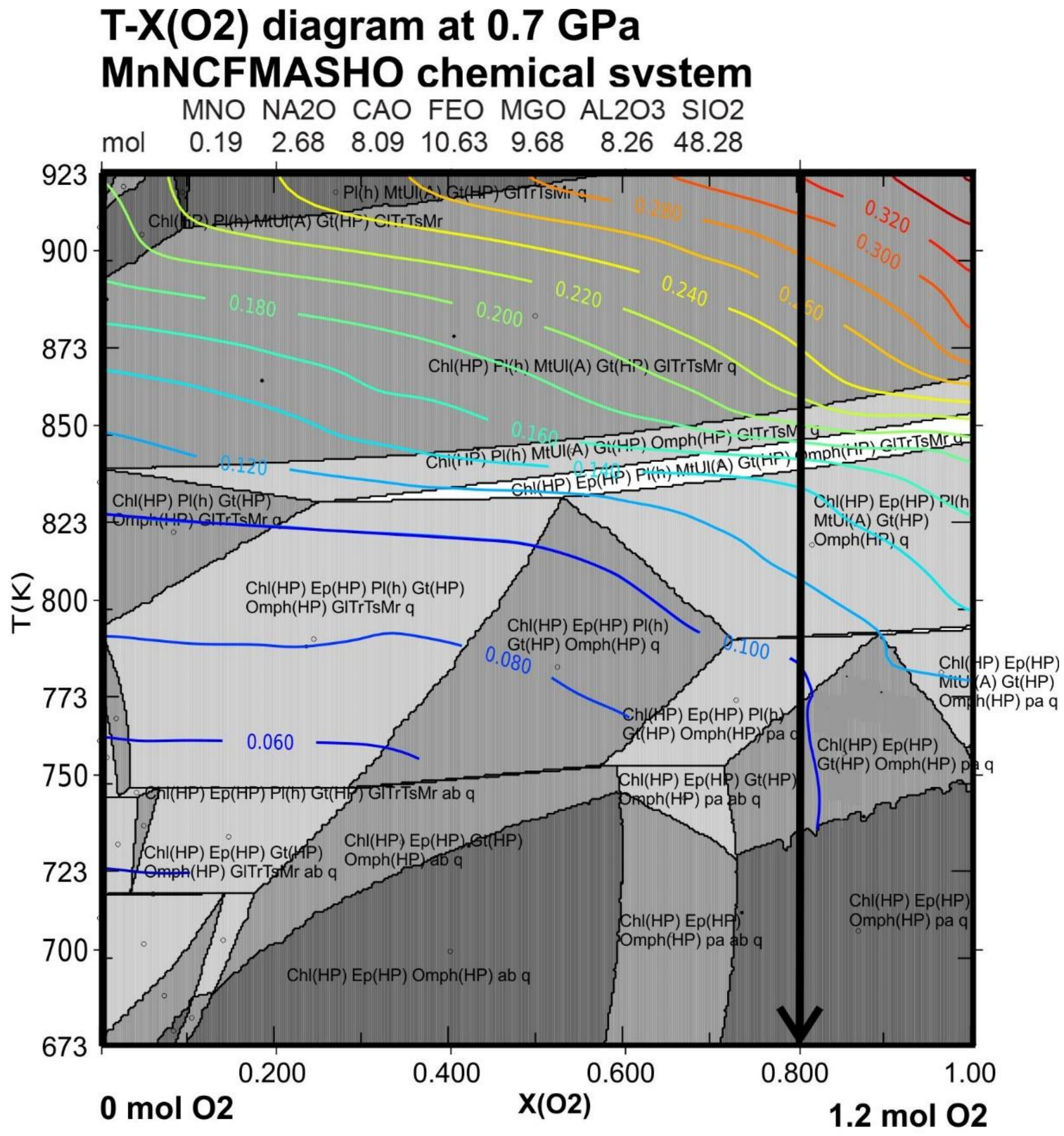

Fig. S1 T–$X(O_2)$ diagram. Evaluation of the ferric iron content and the representative input bulk rock composition for the *P*–*T* diagram for sample P26 in Fig. 11. The starting bulk rock composition obtained from XRF has zero O2 (coordinate 0 on the *T-X* diagram). In the bulk rock composition corresponding to the coordinate 1, 1.2. mol of O2 was added.

The fields with the observed mineral assemblages and their chemical composition was used to obtain the representative bulk rock composition. An important feature of the rock is the presence of magnetite. The right part of the diagram (from 0.5 coordinate) satisfies this observation even along cooling path. The compositional isopleths of XMg value in the garnet fit the best to the region around 0.8 coordinate. The bulk rock composition corresponding to the 0.8 coordinate was thus used as input data for the calculation of the P26 *P-T* diagram.

Ch(HP)= chlorite; Ep(HP) = epidote; Omph(HP) = clinopyroxene; Gt(HP) = garnet; MtUl(A) = magnetite; GlTrTsMr = amphibole; Pl(h) = plagioclase; ab = albite; pa = paragonite; q = quartz.
